# Striatal Interneuron Imbalance in a Valproic Acid-Induced Model of Autism in Rodents Is Accompanied by Atypical Somatosensory Processing

**DOI:** 10.1523/ENEURO.0326-24.2024

**Published:** 2024-12-04

**Authors:** Dayna N. Ibáñez-Sandoval, Ana E. Hidalgo-Balbuena, Ricardo Velázquez Contreras, Nadia Saderi, Gonzalo Flores, Pavel E. Rueda-Orozco, Osvaldo Ibáñez-Sandoval

**Affiliations:** ^1^Departamento de Fisiología y Biofísica, Facultad de Medicina, Universidad Autónoma de San Luis Potosí, San Luis Potosí 78210, México; ^2^Departamento de Neurobiología del Desarrollo y Neurofisiología, Instituto de Neurobiología, UNAM, Querétaro 76230, México; ^3^Facultad de Ciencias, Universidad Autónoma de San Luis Potosí, San Luis Potosí 78295, México; ^4^Instituto de Fisiología, Universidad Autónoma de Puebla, Puebla CP 72570, México

**Keywords:** autism spectrum disorder, sensory processing, striatal interneurons, valproic acid

## Abstract

Autism spectrum disorder (ASD) is characterized by deficits in social interaction and communication, cognitive rigidity, and atypical sensory processing. Recent studies suggest that the basal ganglia, specifically the striatum (NSt), plays an important role in ASD. While striatal interneurons, including cholinergic (ChAT^+^) and parvalbumin-positive (PV^+^) GABAergic neurons, have been described to be altered in animal models of ASD, their specific contribution remains elusive. Here, we combined behavioral, anatomical, and electrophysiological quantifications to explore if interneuron balance could be implicated in atypical sensory processing in cortical and striatal somatosensory regions of rats subjected to a valproic acid (VPA) model of ASD. We found that VPA animals showed a significant decrease in the number of ChAT^+^ and PV^+^ cells in multiple regions (including the sensorimotor region) of the NSt. We also observed significantly different sensory-evoked responses at the single-neuron and population levels in both striatal and cortical regions, as well as corticostriatal interactions. Therefore, selective elimination of striatal PV^+^ neurons only partially recapitulated the effects of VPA, indicating that the mechanisms behind the VPA phenotype are much more complex than the elimination of a particular neural subpopulation. Our results indicate that VPA exposure induced significant histological changes in ChAT^+^ and PV^+^ cells accompanied by atypical sensory-evoked corticostriatal population dynamics that could partially explain the sensory processing differences associated with ASD.

## Significance Statement

One of the main characteristics of patients with autism spectrum disorder (ASD) is the hypo- or hyper-responses to sensory stimuli. Various studies indicate that a possible explanation for these atypical responses is an imbalance in excitation–inhibition modulated by different types of interneurons. In the present work, we provide evidence that a striatal imbalance in ChAT^+^ and PV^+^ levels could partly explain behavioral and somatosensory processing differences associated with the valproic acid-induced ASD model.

## Introduction

Within autism spectrum disorders (ASDs), three important characteristics have been described: (1) differences in social interaction, (2) increased repetitive movements, and (3) cognitive rigidity. Patients with ASD also exhibit sensory dysfunction, characterized by hypersensibility to sound and tactile or visual stimuli (DSM-V; Green et al., 2015; Mikkelsen et al., 2018; Siemann et al., 2020). Although the fundamental mechanisms of sensory hypersensibility are unknown, it has been suggested that circuit-related alterations in the sensory cortex, and particularly a dysfunction of the GABAergic elements of this region (Gogolla et al., 2014; Stoner et al., 2014; Canetta et al., 2016; Q. Chen et al., 2020), play a key role in these differences. Likewise, another area that participates in sensory processing is the striatum (NSt), the main nucleus of the basal ganglia that has been previously suggested as a key participant in ASD (Fuccillo, 2016; Rapanelli et al., 2017a). The NSt processes and integrates inputs from almost the entire cerebral cortex and multiple thalamic regions, including information from sensory and motor areas. Synaptic inputs are processed in medium spiny neurons (MSNs) and different types of GABAergic and cholinergic interneurons (Kawaguchi et al., 1995; Tepper and Bolam, 2004; Tepper et al., 2010, 2018). It has been proposed that the dysfunction of any of these components would lead to poor processing of synaptic inputs and, therefore, aberrant behaviors such as those observed in ASD (Marín, 2012). In this context, previous studies using rats prenatally exposed to valproic acid (VPA-exposed) as a model of ASD have reported anatomical and physiological alterations of MSNs, for example, an increase in both dendritic arborization and dendritic spines in direct pathway MSNs and the opposite in indirect pathway MSNs (Bringas et al., 2013; H. Kim et al., 2022). It has been suggested that these anatomical alterations may be related to changes in MSN excitability (Di et al., 2022; Iezzi et al., 2022). On the other hand, other studies have shown alterations in cholinergic transmission at the cortical (rats/mice VPA-exposed to VPA) and striatal (BTBR mice) levels in different animal models of ASD (J. W. Kim et al., 2014; Karvat and Kimchi, 2014) and a decrease in the number of both cholinergic and parvalbumin-positive (PV^+^) cells in the NSt in the shank mouse ASD model (Filice et al., 2016). Likewise, the depletion of both cholinergic (ChAT) and PV interneurons (Rapanelli et al., 2017b) mimics some autistic features. Research has found that PV^+^ interneurons are fundamental to sculpt MSN ensemble activity and facilitate movement (Gernert et al., 2000; Gittis et al., 2010), and cholinergic interneurons participate in movement control, decision-making, and cognitive processes (Hasselmo and Sarter, 2011; Apicella, 2017; Prado et al., 2017; Gritton et al., 2019). Hence, decreased or dysfunctional activity of these elements could underlie repetitive movements and cognitive rigidity (Xu et al., 2015; Matamales et al., 2016b). To explore these possibilities and the potential contribution of ChAT^+^ and PV^+^ interneurons to sensory hypersensitivity, we studied absolute changes in the number of these cells, as well as somatosensory processing throughout corticostriatal sensory networks associated with a widely used rodent model of ASD by prenatal exposure to VPA (Rodier et al., 1997; J. W. Kim et al., 2014; Maisterrena et al., 2022). We found a significant decrease in both ChAT^+^ and PV^+^ striatal interneuron quantities compared with those of control animals. Moreover, for both cell types, we observed that this decrease was more noticeable in the dorsolateral striatum (DLSt). On the other hand, extracellular recordings of the primary somatosensory cortex (S1) and DLSt in anesthetized animals exhibited exacerbated somatosensory-evoked responses at the single-neuron and population levels. Our data suggest that unbalanced levels of striatal interneurons may be instrumental in sensory process differences associated with autism.

## Materials and Methods

### Animals

Sprague Dawley rats were obtained from the animal facility of the Universidad Autónoma de San Luis Potosí, Mexico. Pregnant rats were housed individually in a temperature-controlled room (24°C) under a 12 h light/dark cycle with food and water *ad libitum*. Long–Evans rats [LE-Tg [Pvalb-iCre] 2Ottc; (*n* = 4); 250–350 g; PV + Cre-Casp] were obtained from the Rat Resource and Research Center at the University of Missouri (MTA-TO; NIMH Ref. No. 2018-0136; Transgenic line 773) and reproduced in our animal facility. To express Caspase 3, the adeno-associated virus pAAV-flex-taCasp3-TEVp was unilaterally injected (1 µl per site of injection) into the DLSt (in mm, AP = 0.6; ML = ±3.5; DV = −4.4). pAAV-flex-taCasp3-TEVp was a gift from Nirao Shah and Jim Wells (Addgene viral prep # 45580-AAV5; http://n2t.net/addgene:45580; RRID: Addgene_45580). All procedures described in the present study were in agreement with the Guide for the Care and Use of Laboratory Animals of the Mexican Council for Animal Care (Norma Oficial Mexicana NOM-062-ZOO-1999), the internal committee (BGFMUASLP-08-20), and the NIH Guide for the Care and Use of Laboratory Animals. All efforts were made to minimize the number of rats used and any possible discomfort.

### Model of ASD in rats

Because autism occurs with a ratio of 3:1 higher incidence in males than that in females (Loomes et al., 2017), the present study was conducted only in male rats. The prenatal exposure to valproic acid (VPA) method was used as described previously (Rodier et al., 1996; Schneider and Przewłocki, 2005; Rinaldi et al., 2008; Markram and Markram, 2010; K. C. Kim et al., 2011; Bringas et al., 2013). In brief, pregnant rats were intraperitoneally injected with VPA (500 mg/kg, single dose; Sigma-Aldrich) or physiological solution at 0.9% of NaCl (PSS), on gestational day 12.5. VPA was dissolved in PSS at a 250 mg/ml concentration. Male offspring were weaned on postnatal day (P) 23. Both groups of animals (i.e., control and VPA-exposed) were kept in separate cages, with a maximum of three animals per cage and in the same conditions as the pregnant rats until their use at the age of P60 for behavior tests or in vivo recordings.

### Behavior tests

Three behavioral paradigms were used to evaluate the ASD model. All behavior protocols were video recorded and analyzed offline, and the “activity test counter” software was used to analyze behavior (Velázquez, 2020). To prevent olfactory cue bias, before each behavioral assay, devices were thoroughly cleaned with a solution containing alcohol 5%, ammonia, and multipurpose cleaner at 5%.

### Social interaction test

The behavioral apparatus consisted of a transparent acrylic box (in cm, 84 long × 35 wide × 25 high) divided into three 28 × 35 cm compartments interconnected by 9 × 7 cm gates (P. Kim et al., 2013; Karvat and Kimchi, 2014). For habituation to experimental conditions, animals (control or VPA) were placed in the central compartment and allowed to freely explore the three compartments for 5 min. Subsequently, a “novel rat” (i.e., a rat that had not been exposed to the experimental animals) was randomly placed in one of the lateral compartments. The “novel rat” was constrained to a transparent Plexiglas chamber (17.5 × 13.5 × 14 cm). We evaluated the following: (1) how much time the animal spent in the novel animal's compartment; (2) the number of contacts with the novel animal, defined as the times that the animal touched or smelled the novel animal; and (3) the interaction time, defined as the amount of time that the animal interacted with the novel animal. The test duration was 10 min.

### Open field test

Individual animals from both groups (control and VPA-exposed) were placed in the center of an acrylic box (70 × 70 × 70 cm) for 20 min (Prut and Belzung, 2003), and the following parameters were analyzed: (1) distance traveled, (2) number of grooming events, and (3) time spent grooming. The last two parameters were indicative of stereotyped behavior (Hartgraves and Randall, 1986; Wu et al., 2019; Li et al., 2024).

### Hole-board test

Animals were placed individually into a black acrylic box (40 × 40 × 30 cm). One of the walls contained a 3 cm diameter hole through which a light stimulus was provided to attract the animal's attention (Rodriguiz and Wetsel, 2006; Bringas et al., 2013). Animals were monitored for 10 min, and the following parameters were evaluated: (1) latency to the first time that the animal passed the head through the hole (head-dipping behavior), (2) frequency of head-dipping, and (3) cumulative head-dipping time.

### Immunohistochemistry

Immunohistochemistry for choline acetyltransferase (ChAT^+^, cholinergic interneurons) or parvalbumin (PV^+^ interneurons) was developed using the peroxidase-diaminobenzidine reaction in samples collected from control and VPA-exposed animals. At the end of each recording session, animals were administered a lethal dose of pentobarbital and transcardially perfused with phosphate buffer saline (PBS, 0.01 M), pH 7.6, followed by 4% paraformaldehyde (PFA; in M): 0.08 Na_2_HPO_4_ and 0.0225 NaH_2_PO_4_. Then, the brains were extracted and kept in PFA overnight before immersion in sucrose solution (30–0.04% NaN_3_ in PBS) to preserve samples. Brains were later sliced (50 μm) with a vibratome (Leica VT1200S) and prepared for immunohistochemical procedures. First, to remove remains of the red globules from the tissue, samples were washed and cleaned with PBS and incubated in a solution with methanol 10%, H_2_O_2_ 0.5% in PBS for 15 min. Then, slices were incubated for 72 h at 44°C with ChAT (anti-Choline Acetyltransferase polyclonal, goat Merck Millipore: AB144P200UL;1:400) or PV (Rabbit Anti-Parvalbumin antibody ab11427; 1:1,200) antibodies in a solution containing 0.01 M Tris-buffered saline (TBS), Triton X-100 0.5% (Sigma-Aldrich), and gelatin 0.25% (Merck KGaA). After this period, the tissue was washed with PBS and incubated with a secondary antibody (ChAT, 1:500, Anti Goat IgG/Donkey, Jackson ImmunoResearch: 705-065-003, biotinylated; PV, 1:1,200, Goat Anti-Rabbit IgG H&L ab207995, biotinylated) for 2 h at room temperature. After that, the slices were washed with PBS and the avidin–biotin complex for 2 h (Kit Vectastain Elite ABC – peroxidase; 1:500). Subsequently, slices were revealed with a solution containing 3,39-diaminobenzidine 0.25% with H_2_O_2_ 0.01% (both Sigma-Aldrich) and nickel ammonium sulfate 10% in TBS for 5 min. Finally, sections were mounted on gelatinized glass slides, dried for 8 h, and dehydrated through a graded series of ethanol, followed by xylene, and covered with epoxy resin in xylol 60% (Hycel lot: 304883). Special care was taken to always process the same number of saline and VPA sections simultaneously, with the same solutions, and in the same multiple 24-well plates.

### Cell counting

Four representative slices containing the NSt (∼1.80 to −0.12 mm with respect to the bregma) were selected for each animal. Sections were examined under a light microscope (Leica DM500) at 10× magnification, and images were captured with a digital camera (Leica ICC50 HD). The perimeter of the NSt was delimitated digitally, and ChAT^+^ and PV^+^ neurons were marked and represented with colored circles. Then, the ventral striatum (VSt), dorsomedial striatum (DMSt), and DLSt were delimitated manually based on anatomical landmarks with reference to the Paxinos and Watson (2014) rat brain atlas. Finally, for each slide and NSt subdivision, the total area was calculated, ChAT^+^ and PV^+^ neurons were counted, and the number of cells per square millimeter was calculated. Digital procedures and quantifications were achieved with Adobe Illustrator CS6 and ImageJ 1.52 K software.

### Anesthetized experiments

Recordings were performed in control, VPA, and PV + Cre-Casp animals anesthetized with urethane (1 g/kg) and mounted on a stereotaxic frame; to maintain optimal anesthetic levels, supplemental doses of urethane (0.15 g/kg) were given when necessary. Silicon probe recordings were performed through a craniotomy (2 × 2 mm) centered at 0.6 mm anterior and 3.7 mm lateral to the bregma. Coordinates were selected based on previous reports demonstrating robust forelimb representations in these S1 and DLSt regions. Neural signals were collected at two depths in S1 (between 0.9 and 1.5 mm below the surface of the brain, corresponding to layers IV and V) and at two to four depths in the DLSt (between 3 and 5 mm below the surface of the brain). Probes were slowly lowered into the recording sites (2 mm/h), and recordings were performed at depths where visual inspection of the signals indicated spiking activity in multiple group channels (probe shanks). Histological confirmation of silicon probe positions in the brain was achieved by applying DiI lipophilic carbocyanine dye (1%; Sigma) to the back of the probes. Somatosensory stimulations (5 ms pulses) were performed on the pads of the forelimb contralateral to the recording site with a bipolar electrode located on the medial (+) and lateral pads (−). Stimulation was given every 5 s as trains of five stimuli (3.3 Hz). Stimulation intensity (∼0.4 mA) was selected based on previous reports (Hidalgo-Balbuena et al., 2019; Peña-Rangel et al., 2021) and set for all animals as the minimal current that produced similar deflections in the local field potential of S1 to a brief tapping on the contralateral forepaw pads with a cotton tip. Immediately after the experiments, animals were injected with a lethal dose of pentobarbital and transcardially perfused; their brains were extracted and processed for histological confirmation or immunohistochemical procedures. Under these conditions, no statistical differences were found in the total number of cells recorded at each depth between control or experimental groups (cells recorded per depth in S1 and striatum: control group, 55 depths, median = 9, 75th = 16, 25th = 5; VPA group, 70 depths, median = 8, 75th = 13, 25th = 5; PV + Cre-Casp group, 27 depths, median = 9, 75th = 15, 25th = 6; K–W, *X*^2^ = 0.97; *p* = 0.61).

### Electrophysiological data acquisition and processing

Sixty-four-channel silicon probe arrays (NeuroNexus, Buzsaki64) were used in all recordings. Wideband (0.1–8,000 Hz) neurophysiological signals were amplified 1,000 times via the Intan RHD2000-series amplifier evaluation system. Spike sorting was performed semiautomatically using the clustering software KlustaKwik (http://klustakwik.sourceforge.net) and the graphical spike sorting application Klusters (http://klusters.sourceforge.net; Hazan et al., 2006).

### Analysis of neural data

#### Sensory-evoked patterns

The principal component analysis (PCA)/silhouette-based method was used as reported in Peña-Rangel et al. (2021) and Pimentel-Farfan et al. (2022). In brief, PCA was applied to the *z*-scored cutaneous stimulation-evoked patterns of each recorded neuron in a time window of 1.5 s aligned to the first stimulus of the train. To assign cells to specific clusters with similar characteristics, *k*-means was applied to the first three principal components (PCs). To obtain the best classification, the process was repeated 1,000 times with projections ranging from three to eight clusters. Each projection was scored with the silhouette method, and the projections with the highest silhouettes scores were selected. This procedure was also applied to extract autocorrelogram shapes in the S1 and DLSt and for spike-wave shapes in the DLSt.

#### Statistics

For behavioral statistical analysis, the Shapiro–Wilk test was applied to assess normal distribution, and an unpaired *t* test was used for group comparisons, and the data is reported as the mean ± SEM. For anatomical data, groups were compared using an unpaired *t* test. In general, non-normal distributions were observed in electrophysiological data; hence, nonparametric statistics were used for this type of data. For most of the cases, Kruskal–Wallis (K–W) and Bonferroni’s post hoc test were utilized for multiple comparisons. The Wilcoxon test was applied as indicated in the text.

## Results

### Behavioral and cognitive assessments in VPA-exposed animals

**Figure 1.**
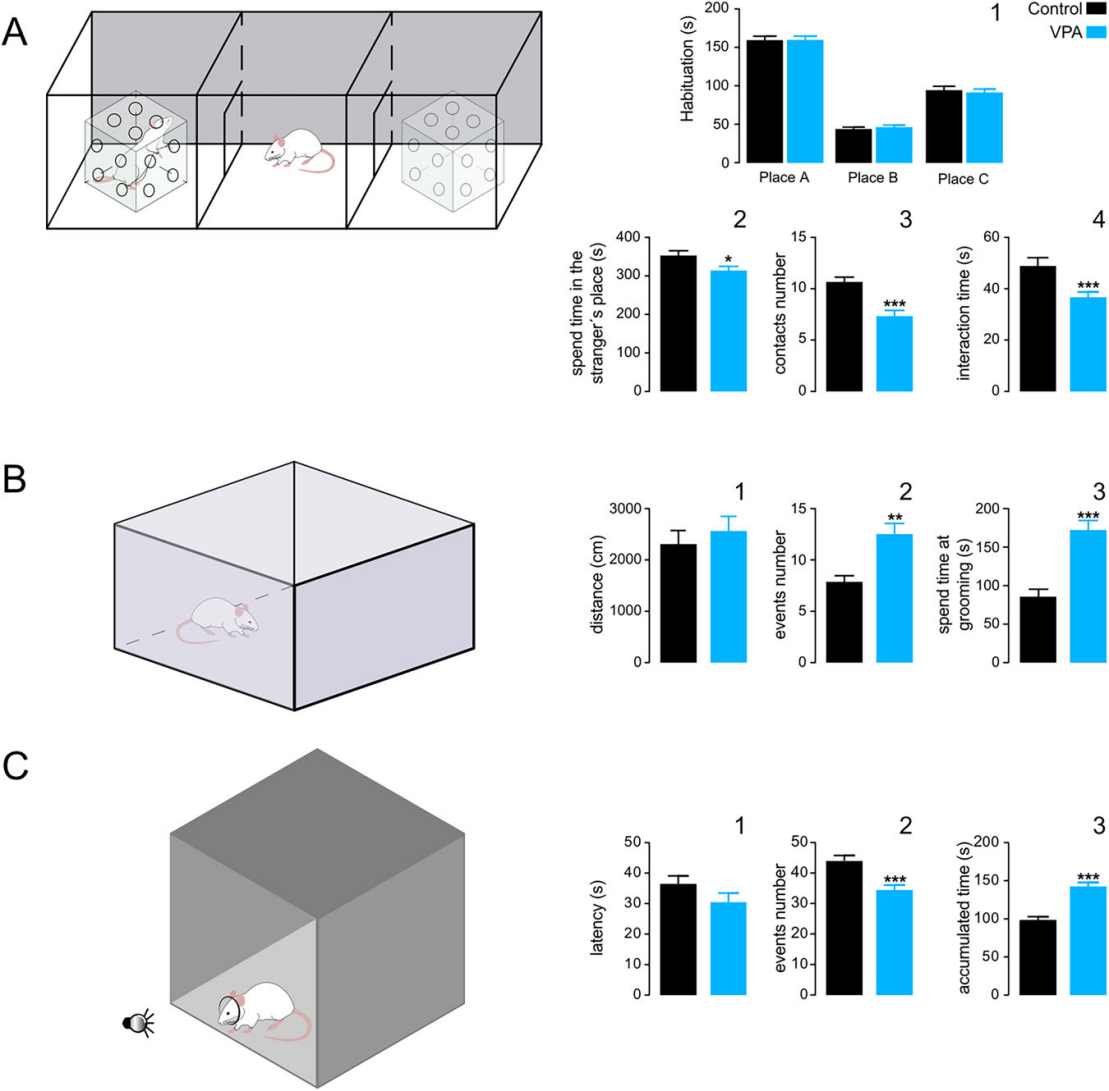
VPA-exposed animals exhibited the main characteristics of ASD. ***A***, Schematic representation of the box with three chambers (left). The “test rat” was placed in the center compartment and the novel rat was placed in a lateral compartment. The average times spent in each compartment during the habituation session (***A1***), the time spent in the “novel compartment” during the experimental session (***A2***), the number of contacts with the novel animal (***A3***), and the total interaction time with the novel animal (***A4***) are displayed in the right bar plots summarizing eighteen experiments which show that VPA animals exhibited significantly lower levels of interaction with the novel animal than with the control group. ***B***, Schematic representation of the open field apparatus (left). Total traveled distance (***B1***), number of events (***B2***), and total time spent in grooming behavior (***B3***) are summarized in the right bar blots showing that VPA animals expressed significantly increased levels of stereotyped behavior than the control animals. ***C***, Schematic representation of the hole-board apparatus (left). The latency to the first entrance (***C1***), the total number of crossings through the hole (***C2***), and the total accumulated time spent inside the hole (***C3***) are displayed in the right panels; these findings suggest increased attentional levels in the VPA animals. *p < 0.05; ***p < 0.01; ***p < 0.001.

One of the main characteristics of ASD patients is the lack of social interaction (Boraston et al., 2007; Liu et al., 2019; Bos et al., 2020; Wang et al., 2020), which has also been reported in different rodent models of ASD, such as the VPA-based model or genetic-based models (BTBR; P. Kim et al., 2013; J. W. Kim et al., 2014, 2017; Karvat and Kimchi, 2014). Here we evaluated social interaction at P56–60 (control offspring *n* = 18 of *N* = 8 pregnant nontreated rats; VPA offspring *n* = 18 of *N* = 10 pregnant VPA-exposed rats). Our data show that the VPA animals spent significantly less time in the compartment with the “novel rat” than the control animals (control 352.8 ± 12.26 s vs VPA 314.1 ± 10.68 s; *p* = 0.0231; Fig. 1*A2*). Moreover, compared with the control group, VPA animals showed significantly fewer contacts (control 10.67 ± 0.46 vs VPA 7.33 ± 0.57; *p* < 0.0001; Fig. 1*A3*) and a significantly shorter cumulative time of interaction (control 49.31 ± 2.78 s vs VPA 37.15 ± 1.56 s; *p* = 0.0006; Fig. 1*A4*) with the novel rat. Importantly, both groups of animals exhibited similar times in each compartment during the habituation period, discarding potential differences related to the baseline levels of exploration (Fig. 1*A1*).

Another principal feature of ASD is the presence of stereotyped behaviors. To explore whether this behavior was present in our ASD model, VPA and control animals were evaluated in an open field test (Fig. 1*B*; Prut and Belzung, 2003; D. Chen et al., 2019). Consistent with our previous quantification (Fig. 1*A4*), both groups of animals showed similar traveled distances (control 2,308 ± 266.1 cm vs VPA 2,563 ± 285 cm, ns; Fig. 1*B1*). However, compared with the control group, VPA animals exhibited a significant increase in the frequency (control 7.86 ± 0.6218 vs VPA 12.43 ± 1.142; *p* = 0.0014; Fig. 1*B2*) and duration of grooming behavior (control 84.96 ± 9.957 vs VPA 170.7 ± 12.21; *p* = 0.0001; Fig. 1*B3*), a commonly accepted indicator of increased stereotyped behavior.

Finally, another trait associated with ASD is cognitive rigidity. It has been proposed that one possible way to assess cognitive rigidity in rodents is by evaluating the amount of time that animals spend responding to a salient stimulus. Increased times may be indicative of exacerbated or obsessive attention, also known as attention reorienting deficit (Markram and Markram, 2010; Orekhova and Stroganova, 2014). To evaluate this behavior, we used a variation of the hole-board test (modified from Rodriguiz and Wetsel, 2006; Fig. 1*C*). Our data show that control and VPA animals exhibited similar latencies to the first head-dipping behavior (Fig. 1*C1*). However, VPA animals displayed significantly fewer events (control 44.33 ± 1.35 vs VPA 34.83 ± 1.09; *p* < 0.0001; Fig. 1*C2*) and accumulated more time with the head hole than control animals (control 99.77 ± 2.74 vs VPA 144 ± 3.41; *p* < 0.0001; Fig. 1*C3*), suggesting that the VPA-exposed animals maintained a higher level of attention to the light stimulus.

These results show that our VPA model produced behavioral traits consistent with those previously reported in children with ASD and environmental and genetic animal models, such as impaired social interaction (Karvat and Kimchi, 2014; Franchini et al., 2017; Supekar et al., 2018), increased behavioral stereotypy (J. W. Kim et al., 2017; Hirsch et al., 2018), and attention re-reorienting deficit (Orekhova and Stroganova, 2014). It should be noted that all behavioral tests by themselves present limitations. For example, in our version of the social interaction test, the results may be interpreted as a general lack of interest in exploration and not as a specific effect in the social domain. On the other hand, the results are limited to the interaction with a novel rat and not to social preference, as could also be measured by introducing a familiar versus a novel animal (J. W. Kim et al., 2017).

### Decrease of striatal ChAT^+^ and PV^+^ cells in VPA-exposed animals

Previous reports in both, shank mice and rats prenatally exposed to VPA, have shown that animal models of ASD exhibit a decrease in the number of striatal PV+ cells (Filice et al., 2016; Lauber et al., 2016; Rapanelli et al., 2017a) and that the depletion of both cholinergic and PV+ interneurons in the NSt of male mice (Rapanelli et al., 2017b) mimics some characteristics of ASD. To confirm these possibilities and explore the significance of these interneurons in striatal functionality, first we developed immunohistochemical quantifications for both types of interneurons in control and VPA animals.

**Figure 2.**
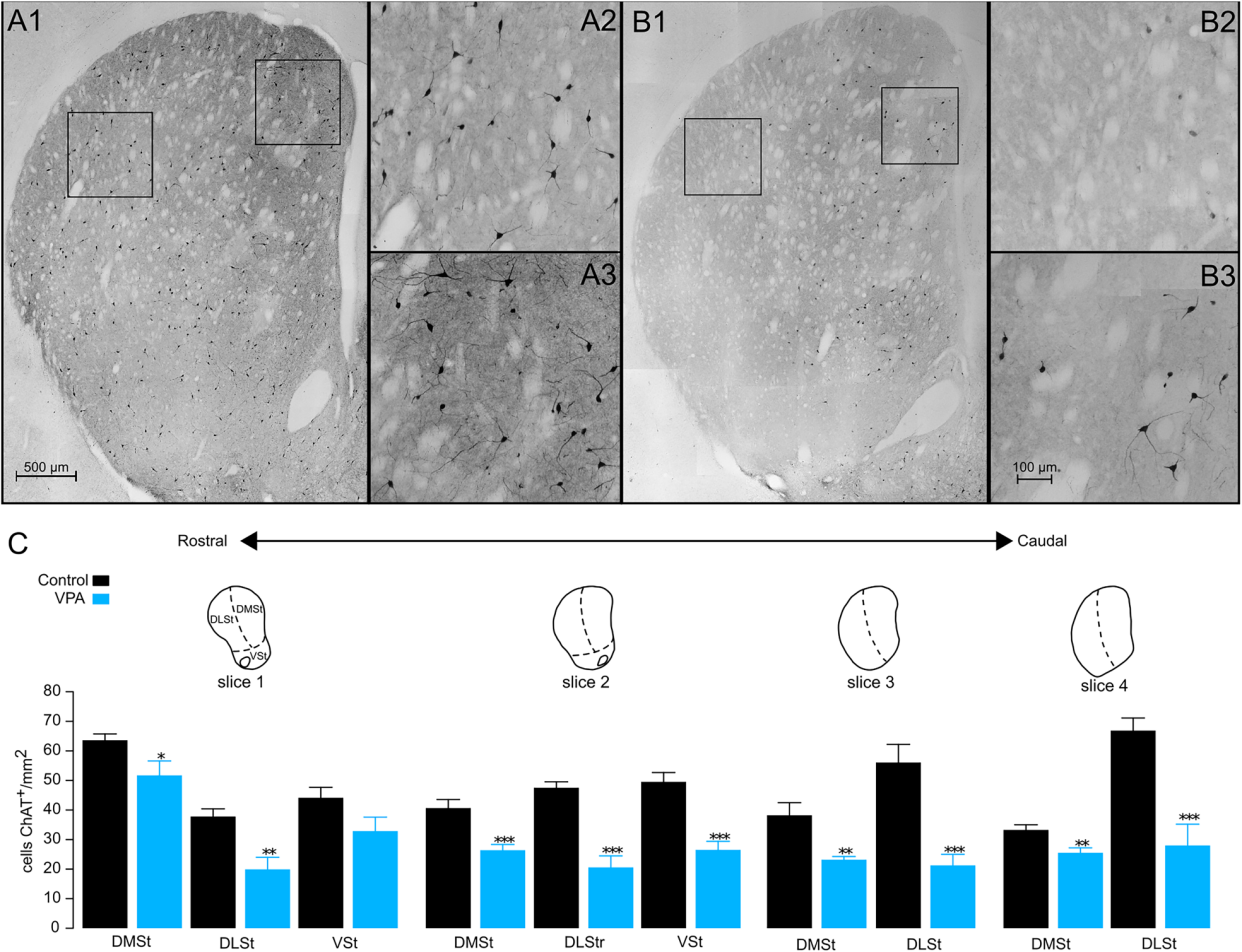
Decrease of striatal ChAT^+^ cells in VPA animals. Representative microphotographs of the NSt from control (***A***) and VPA (***B***) animals. Note the homogeneous distributions of ChAT^+^ cells throughout the NSt in control animals (***A***) and a significant reduction of ChAT^+^ cells in VPA animals (***B***). Formal quantification of immune-positive cells was performed in the dorsomedial (***A2***, ***B2***) and dorsolateral (***A3***, ***B3***) regions of the striatum, indicated by squares in ***A1*** and ***B1***. The reduction of ChAT^+^ cells was greater in the dorsolateral striatum (DLSt; compare ***A2*** and ***B2***) than in the dorsomedial striatum (DMSt; see ***A3*** vs ***B3***). ***C***, Summary plots of seven brains from each group of animals (control, black; VPA, blue). *p < 0.05; **p < 0.01; ***p < 0.001.

To analyze the striatal cholinergic system, we used antibodies against the enzyme choline acetyltransferase (ChAT^+^) that is present in all cholinergic cells (Bolam et al., 1984). A detailed inspection of the brain slices showed a homogeneous distribution of ChAT^+^ interneurons throughout the entire NSt of control animals (Fig. 2*A1–3*). In contrast, ChAT^+^ interneurons in VPA animals were scarce, and a formal statistical comparison revealed a general significant decrease of 38.2% (control, 2,335 ± 93.98 cells vs VPA, 1,443 ± 187.1 cells; *p* = 0.0011; Fig. 2*B1–3*). Furthermore, VPA sections showed a fainter mark in neuropil than the control sections (Fig. 2, compare *A1,2*, *B1,2*). Then we explored whether the decrease was a general phenomenon throughout the NSt or if it was exacerbated in specific regions. We observed that the most important decrease was localized at the level of the DLSt (56.9%), followed by the VSt (36.81%) and DMSt (27.84%; Fig. 2*C*, Table 1). In addition, we observed that the decrease of ChAT^+^ cells in the NSt was more pronounced as we moved through the slices from rostral to caudal. Finally, in the control animals, we also noticed that the density of ChAT^+^ cells in the DMSt decreased in the caudal direction, whereas in the DLSt it followed the opposite pattern (Fig. 2*C*, Table 2).

**Table 1.** Descriptive statistics of the total density of ChAT^+^ and PV^+^ cells/mm^2^ for striatal regions

Region	ChAT^+^ cells/mm^2^			PV^+^ cells/mm^2^		
	Mean ± SEM		*p*	Mean ± SEM		*p*
	Control	VPA		Control	VPA	
DLSt	50.32 ± 2.460	21.69 ± 2.139	<0.0001	28.81 ± 0.7142	14.38 ± 0.6615	<0.0001
DMSt	45.25 ± 2.266	32.65 ± 2.3111	0.0002	27.04 ± 0.5762	16.76 ± 0.7669	0.0002
VSt	46.88 ± 2.3777	29.62 ± 2.728	<0.0001	33.20 ± 1.022	23.61 ± 4.410	0.0457

**Table 2.** Descriptive statistics of ChAT^+^ cells/mm^2^ for slices

		Slice 1			Slice 2			Slice 3			Slice 4		
		Mean ± SEM		*p*	Mean ± SEM		*P*	Mean ± SEM		*p*	Mean ± SEM		*p*
DLSt	Control	37.84	2.595	0.0010	47.54	2.055	<0.0001	56.10	6.142	<0.0001	66.90	4.254	0.0003
	VPA	19.98	4.071		20.63	3.858		21.30	3.723		28.03	7.190	
DMSt	Control	63.62	2.117	0.0349	40.70	2.83	0.0003	38.25	4.253	0.0021	33.30	25.55	0.0069
	VPA	51.79	4.873		26.46	1.899		23.25	1.064		1.725	1.673	
VSt	Control	44.16	3.552	ns	49.59	3.119	<0.0001						
	VPA	32.91	4.709		26.55	2.862							

**Figure 3.**
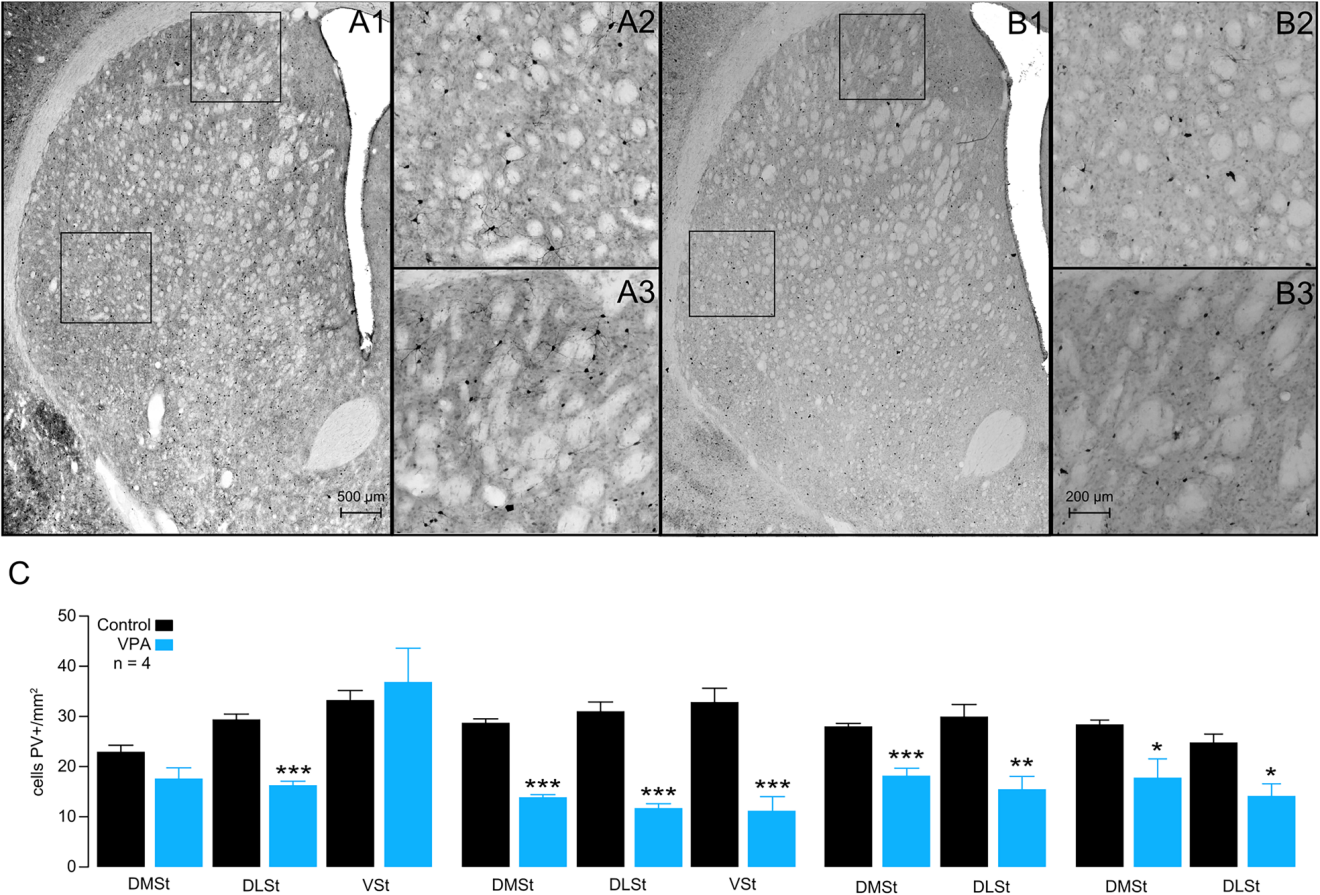
Decrease of striatal PV^+^ cells in VPA-exposed animals. Representative microphotographs of the NSt from control (***A***) and VPA (***B***) animals showing the distribution of PV^+^ cells. Formal quantification of immune-positive cells was performed in the dorsomedial (***A2***, ***B2***) and dorsolateral (***A3***, ***B3***) regions of the striatum, indicated by squares in ***A1*** and ***B1***. The reduction in the PV labeling of cells was greater in the DLSt (see ***A2*** vs ***B2***) than in the DMSt (see ***A3*** vs ***B3***). ***C***, Summary plots of seven brains from each group of animals (control, black; VPA, blue). *p < 0.05; **p < 0.01; ***p < 0.001.

On the other hand, another important element of the NSt microcircuit that has been reported to be affected in neurodevelopmental disorders is the PV^+^ interneurons (Kataoka et al., 2010; Filice et al., 2016; Lauber et al., 2016; Rapanelli et al., 2017a). According to our immunohistochemical quantifications, VPA animals exhibited a 42.5% decrease in the total number of PV^+^ interneurons along the NSt (control, 3,617 ± 207.3 vs VPA, 2,080 ± 88.67; *p* = 0.0024; Fig. 3*A*,*B*). Similar to the observations in ChAT^+^ cells, the decrease was greater in the DLSt (50.8%) than in the DMSt (38%) or VSt (28.9%; Table 1). When analyzing PV^+^ interneurons in the rostrocaudal axis, we found that the three subdivisions (VSt, DMSt, and DLSt) presented the lowest quantities in the medial regions (Table 3, Fig. 3*C*).

**Table 3.** Descriptive statistics of PV^+^ cells/mm^2^ for slices

		Slice 1			Slice 2			Slice 3			Slice 4		
		Mean ± SEM		*P*	Mean ± SEM		*p*	Mean ± SEM		*p*	Mean ± SEM		*p*
DLSt	Control	29.46	1.038	<0.0001	31.07	1.817	<0.0001	29.99	2.377	0.0059	24.81	1.658	0.0108
	VPA	16.31	0.804		11.76	0.868		15.51	2.534		14.18	2.403	
DMSt	Control	22.97	1.312	0.0781	28.76	0.757	<0.0001	28.03	0.600	0.0008	28.4	0.872	0.0328
	VPA	17.64	2.145		13.92	0.502		18.22	1.473		17.81	3.735	
VSt	Control	33.26	1.934	ns	32.88	2.731	0.0014						
	VPA	36.91	6.692		11.22	2.788							

### Baseline neuronal dynamics in S1 and DLSt

**Figure 4.**
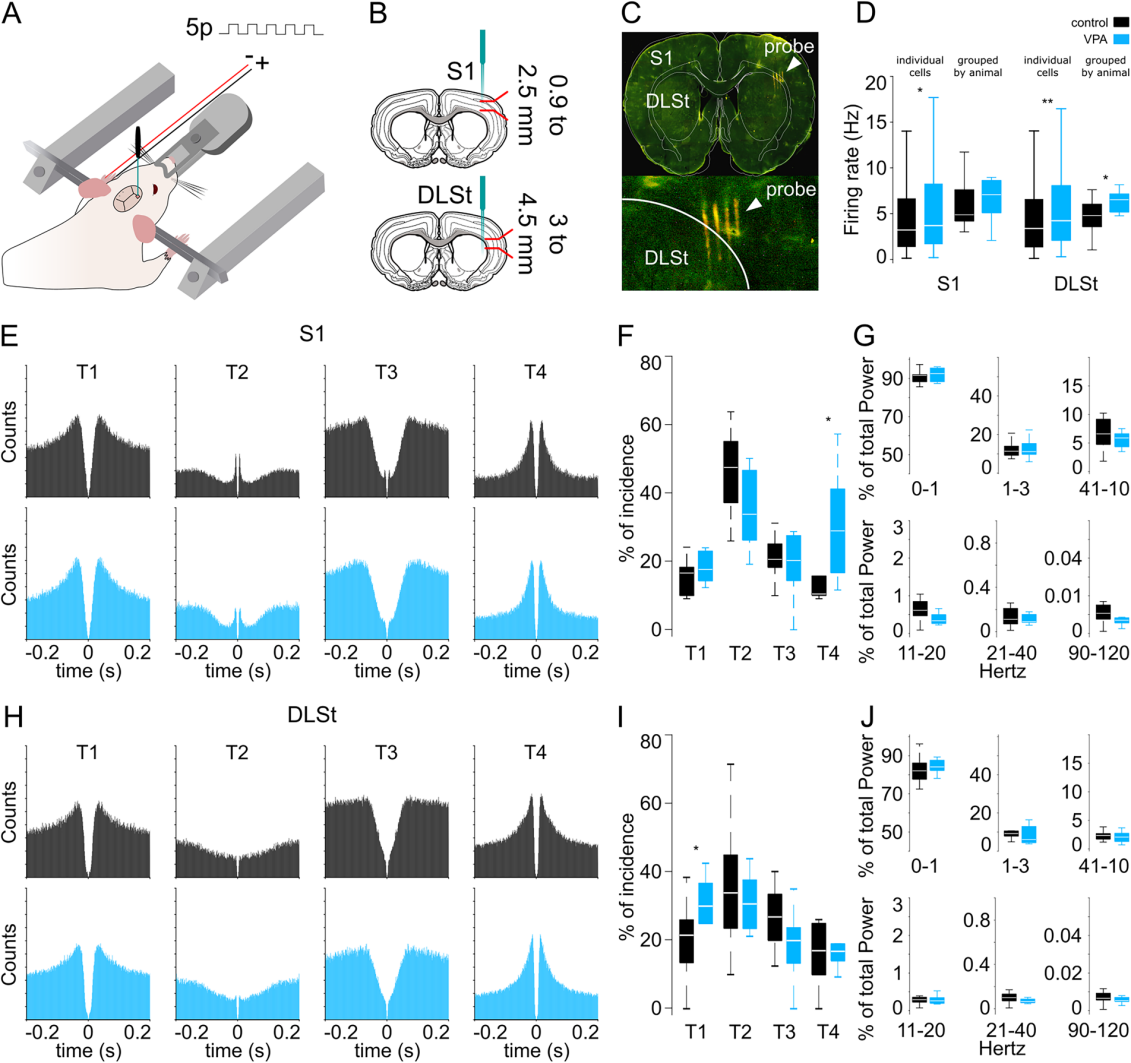
Spontaneous activity in S1 and DLSt neurons in anesthetized animals. ***A***, Schematic representation of the acute preparation for recordings in deeply anesthetized rats fixed on a stereotaxic frame. Stimulation consisted of 50 trains of five 5 ms electrical pulses (3.3 Hz) applied to the forepaw contralateral to the recording sites in S1 or DLSt (***B***, ***C***). For the rest of the figure, control and VPA groups are presented in black and blue, respectively (Swanson, 2018). ***D***, Firing rates for all cells recorded and grouped by animal in the S1 and DLSt. ***E***, ***H***, Average autocorrelograms for all cells belonging to the specific subgroups recorded in the S1 (***E***) and DLSt (***H***). ***F***, ***I***, Percentage of occurrence of each autocorrelogram type for neurons recorded in the S1 (***F***) and DLSt (***I***; K–W and Bonferroni’s post hoc test). ***G***, ***J***, Relative contribution of specific band frequencies to the total autocorrelogram power for neurons recorded in the S1 (***G***) and DLSt (***J***). Wilcoxon rank sum test, all *p* values >0.07. Boxplots depict median and the 25th and 75th percentiles; significant differences are indicated by asterisks.

The decrease in striatal ChAT^+^ and PV^+^ cells associated with the ASD model suggests an important disturbance in the striatal microcircuit functionality and, hence, inadequate processing of both motor and somatosensory cortical inputs. To explore this possibility, we performed extracellular recordings on urethane-anesthetized animals (Fig. 4*A*), a preparation in which somatosensory-evoked signals can be reliably recorded in S1 and DLSt (Hidalgo-Balbuena et al., 2019; Báez-Cordero et al., 2020; Peña-Rangel et al., 2021). Neural recordings were performed in VPA and control adult rats (P70–90) in S1, where a decrease in GABAergic interneurons has been reported in ASD models (Lauber et al., 2016; Q. Chen et al., 2020) and in the DLSt, where we observed the most important decrease in ChAT^+^ and PV^+^ interneurons (Fig. 4*A*,*B*). Sensory-evoked responses were obtained by applying trains of five stimuli (3.3 Hz, 5 ms/stimulus) to the palm of the forelimb contralateral to the recording site (Fig. 4*A*; see Materials and Methods).

We analyzed the activity of 610 well-isolated neurons from S1 and 945 from the DLSt of 26 animals (control *n* = 12; VPA *n* = 10; and PV + Cree-Casp *n* = 4), 295 and 340 neurons were collected from S1 and the DLSt, respectively. From the VPA animals, 315 and 388 neurons were obtained from the S1 and DLSt, respectively. And from the PV + Cre-Casp animals, 217 neurons were recorded in the DLSt. Neurons recorded from VPA animals presented slightly but significantly higher baseline firing rates than neurons recorded from control animals in both structures. These differences were observed when comparing all individual neurons or divided by recorded animal (Fig. 4*D*). To explore possible differences in intrinsic properties between groups, we analyzed spontaneous oscillatory patterns in the spiking activity of individual neurons. By using a PCA-based method to classify the shape of individual autocorrelograms (Peña-Rangel et al., 2021), we identified four prevalent patterns in both groups in the S1 (Fig. 4*E*) and DLSt (Fig. 4*H*). These patterns were similarly distributed in both groups in the S1, but with a higher prevalence of pattern type 4 for the VPA group (Fig. 4*F*; K–W, *X*^2^ = 37.1; DF = 7; *p* < 0.001). On the other hand, a higher representation of pattern type 1 was observed in the DLSt of VPA animals (Fig. 4*I*; K–W, *X*^2^ = 19.02; DF = 7; *p* = 0.008). This pattern corresponds to neurons with tonic firing rates. Then, for each neuron we analyzed the relative oscillatory power of the autocorrelogram at different frequencies and found no significant differences between groups in either structure (Fig. 4*G*,*J*).

**Figure 5.**
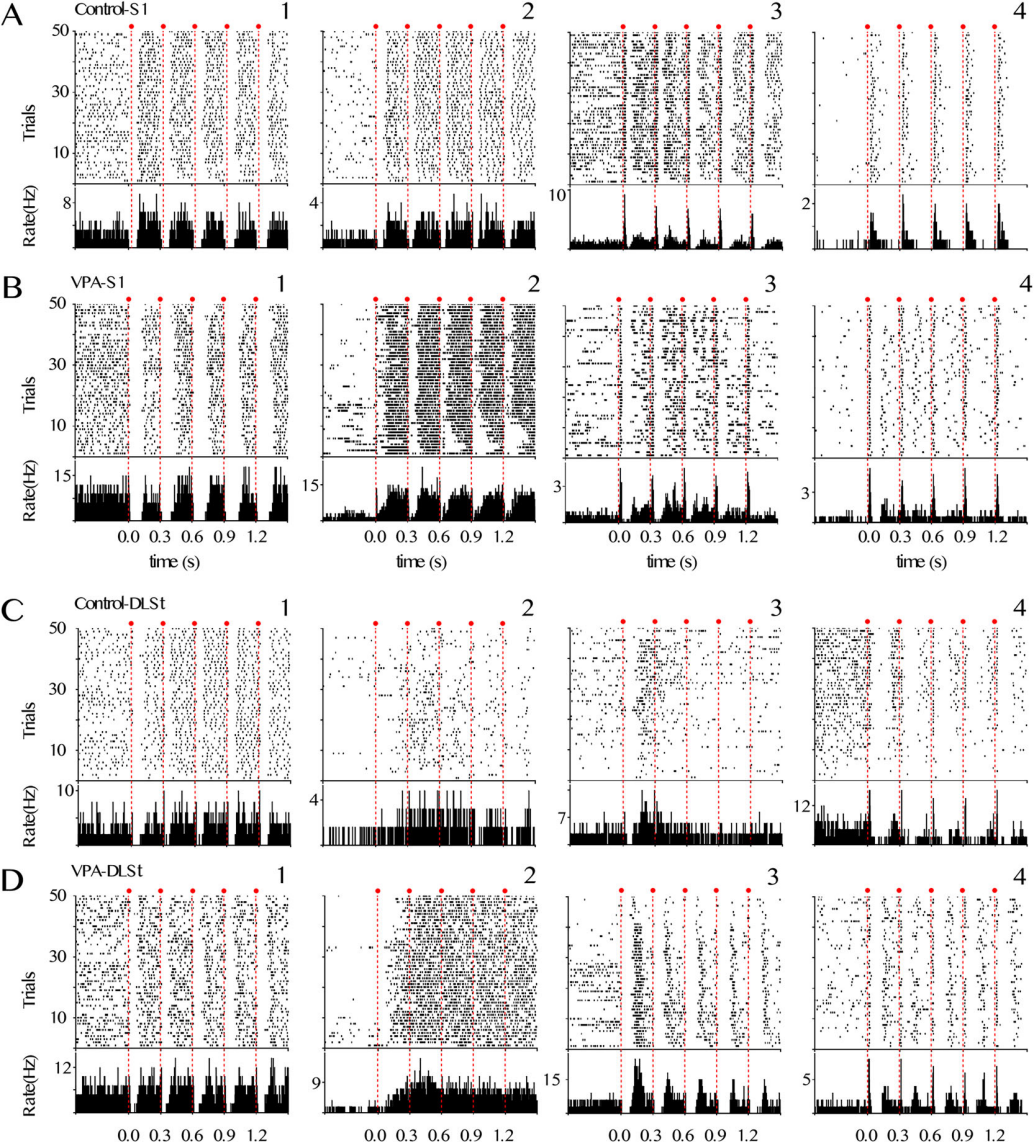
Sensory-evoked response in S1 in VPA animals. ***A–D***, Spike rasters (top) and average peri-event histograms (bottom) for representative neurons with different response patterns recorded in the S1 (***A***, ***B***) and DLSt (***C***, ***D***) of control (***A***, ***C***) and VPA (***B–D***) animals; red dots and dashed lines indicate somesthetic stimulations. Four different representative cells (patterns) are depicted for each structure and condition.

The slight increase in baseline firing rates suggests a greater excitability in the cortical and striatal networks of VPA animals (Fig. 4*D*). To explore the possible consequences of these differences, we evaluated somatosensory processing evoked by cutaneous stimulation of the forelimb contralateral to the recording site (Fig. 4*A*). S1 and DLSt neurons in both control and VPA animals showed different patterns of reliable sensory-evoked responses, as depicted in the raster plots of multiple representative neurons (Fig. 5*A*,*B*). Consistent with previous reports (Hidalgo-Balbuena et al., 2019), in all conditions, many neurons presented complex patterns of activation, being the most salient sharp and short-latency responses to each stimulus of the train. Other neurons presented a complex response, which consisted of a short-latency increase in the activity followed by a transient pause followed by a rebound. Finally, some neurons presented a brief and short-latency pause in their activity.

### Somatosensory-evoked neural activity in S1

**Figure 6.**
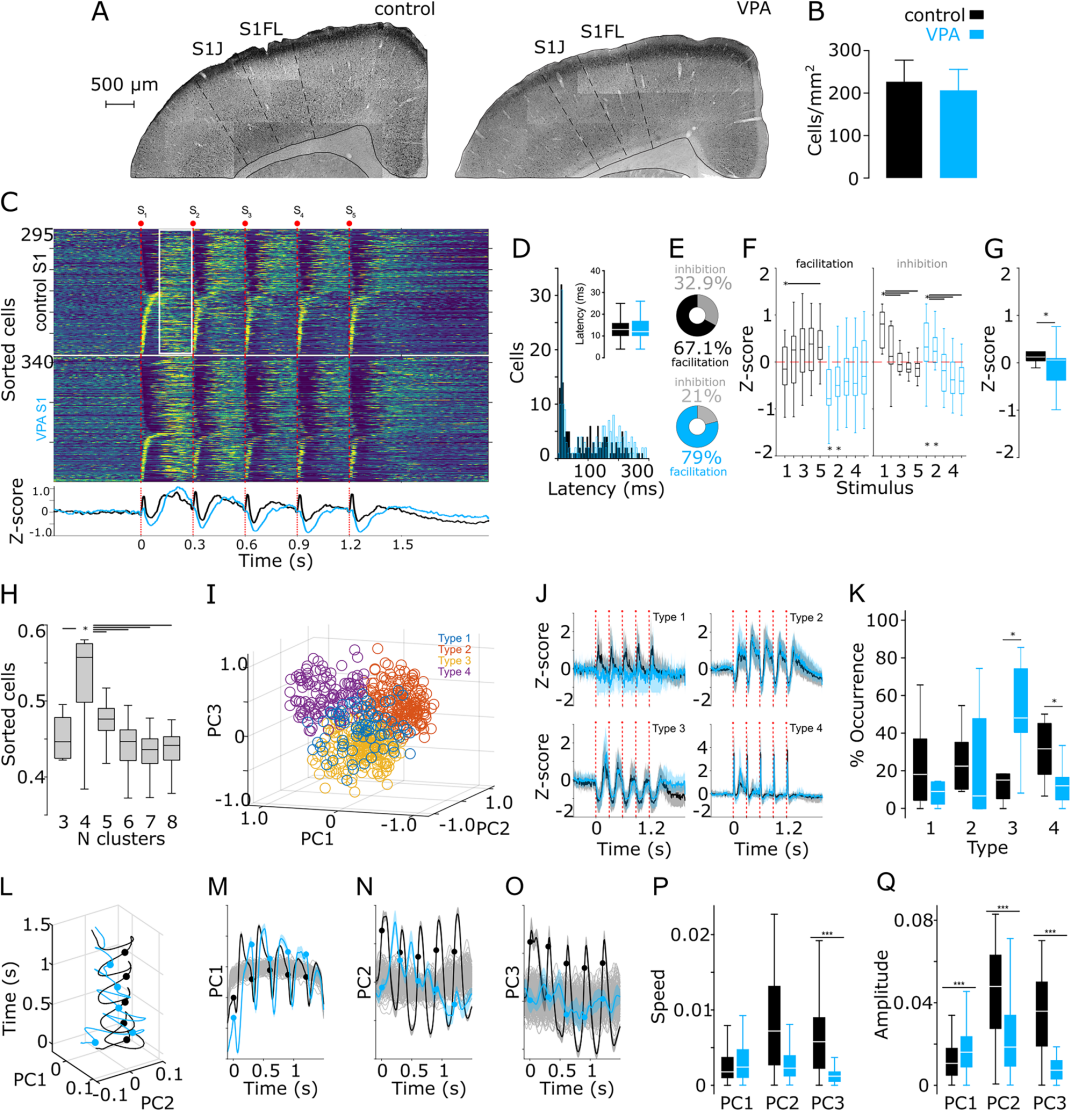
S1 population dynamics in control and VPA animals. ***A***, Representative microphotographs of S1 from control (left) and VPA (right) animals showing the distribution of PV^+^ cells. ***B***, Formal quantification of immune-positive cells in S1. For the entire figure, control and VPA groups are presented in black and blue, respectively. ***C***, Averaged firing rates for cells recorded in S1 of control (top) and VPA (bottom) animals, expressed as *Z*-score (range value, −1 to 3) and sorted according to the time they reached the highest (bottom to top) or lowest (top to bottom) activity after somesthetic stimulation (indicated by red dots and lines). Bottom traces represent averaged histograms of the population response for all cells recorded. ***D***, Response latencies to the first stimulus of the train; latencies under 50 ms are compared in the inset panel. ***E***, Percentage of neurons classified as facilitating (solid color) or depressing (gray) based on their linear growth (or decay) profile of adaptation to the progression of the stimulation train. ***F***, Comparison of the response amplitude for the long-latency increase component of the sensory-evoked responses (example with squared area in ***C***). Top asterisk and lines indicate intra-group statistical differences between the first stimulus of the train (asterisk) and the corresponding subsequent stimuli (joined by lines). Asterisks at the bottom represent inter-group statistical differences for the corresponding stimulus. ***G***, Amplitude of the average general population's joint response to the five stimuli of the trains (S1–S5). ***H***, Silhouette values for 1,000 iterations in 3–8 *k*-means projections from the PCA on the peri-event histograms of the sensory-evoked spiking activity. ***I***, Best PC projection corresponding to 4 clusters (color coded). ***J***, Averaged sensory-evoked responses for cells classified as part of specific sensory-evoked patterns in control and VPA animals. Solid lines and shaded areas represent the median and the 25th and 75th percentiles, respectively. ***K***, Percentages of cells belonging to the different classified patterns in ***J***. ***L***, Low-dimensional PC-3D projection of the population activity in S1 evoked by 50 stimulation trains for control and VPA recordings. PC-2D projections for PCs 1–3 (***M–O***) versus time. Gray lines represent 1,000 surrogate PC projections obtained from shuffling spike trains. Comparison of the trajectory speed (***P***) and amplitude (***Q***) for each PC in control and VPA recordings. For corresponding panels, boxplots indicate median and 75th and 25th percentiles. Statistical differences are indicated by asterisks and lines joining specific comparisons and were obtained with K–W (as indicated in the main text) and the Bonferroni’s post hoc test (*p < 0.05; ***p < 0.01).

PV^+^ cell levels in control and VPA animals did not differ significantly, which is inconsistent with our anatomical observations in the NSt (Fig. 6*A*,*B*). To further analyze the network dynamics of control and VPA networks before, during, and after the somatosensory stimulation train (SST), we calculated the *Z*-score values of the evoked spiking activity (Fig. 5*A*,*D*) aligned to the first stimulus of the SST. Following the trend observed in the representative neurons (Fig. 5), both groups presented a complex population response consisting of subpopulation neurons that increased or decreased their spiking activity in a transitory manner (Fig. 6*C*). A visual inspection of the data revealed that both experimental groups (control and VPA) had a similar general average response shape consisting of a short-latency increase in activity, followed by a transitory pause, and finally a second long-latency increase or “rebound” in activity. This pattern was repeated for each stimulus of the train (Fig. 6*C*). However, the reduced activity component was stronger in the VPA population than in the control population, and as the stimulus train progressed, the short-latency increases appeared to be smaller (Fig. 6*C*, bottom panel). To confirm these observations, we first analyzed the latency of the increased activity and found no differences between groups (Fig. 6*D*). Then, we aimed to analyze the amplitude of the rebound phase of the response; for this, we averaged the activity of the 200 ms windows starting 80 ms after the onset of each stimulus of the train, rendering five values (one for each stimulus) per cell (Fig. 6*C*, white square). Because the train stimulation could induce facilitation or depression on different neurons, we first calculated the linear growth (or decay) rate of the response amplitude (200 ms window starting 80 ms after stimulus onset) for each neuron and divided the population according to units that presented facilitation or depression. We found that VPA animals had a higher proportion of facilitating units (Fig. 6*E*), but both groups displayed similar values of linear growth or decay (growth: control, 25th = 0.03, 50th = 0.11, 75th = 0.23; VPA, 25th = 0.04, 50th = 0.11, 75th = 0.23; K–W, *X*^2^ = 2.41, *p* = 0.29; decay: control, 25th = −0.33, 50th = −0.19, 75th = −0.08; VPA, 25th = −0.28, 50th = −0.17, 75th = −0.07; K–W, *X*^2^ = 0.47, *p* = 0.78). Then we calculated the amplitude of the responses of facilitating and depressing units. We found clear facilitating and depressing dynamics in both groups. We identified these dynamics by the significant differences between the amplitude of the first stimulus of the train and the following amplitudes (Fig. 6*F*, top asterisk and lines). However, when we compared the amplitudes of the control and VPA animals, we found that for some train stimuli, the VPA group displayed lower amplitudes (Fig. 6*F*, bottom asterisks), suggesting slightly different population dynamics (facilitating units K–W, *X*^2^ = 13.83, DF = 9; *p* = 0.017; inhibiting units, K–W, *X*^2^ = 29.75, DF = 9; *p* < 0.001). These were more evident when we averaged all stimulus responses and found that VPA animals presented a significantly larger inhibitory component than the control group (Fig. 6*G*; Wilcoxon test, *p* = 0.01).

Then, to further explore potential evoked response differences between groups, we aimed to classify the response patterns of individual neurons and assess their prevalence in the population. As previously reported (Peña-Rangel et al., 2021), we applied a PCA/silhouette-based method to classify the average response shape of each neuron as part of clusters with similar characteristics (see Materials and Methods). We tested multiple *k*-means projections ranging from three to eight cluster shapes and found that the projection with the highest silhouette values (indicating higher intra-cluster cohesion and inter-cluster separation; Fig. 6*H*; K–W, *X*^2^ = 4,375, DF = 5; *p* < 0.001) consisted of four clusters (Fig. 6*I*) corresponding to four distinct S1 cortical patterns (Fig. 6*J*) expressed in both control and VPA animals. However, when we analyzed the prevalence of each pattern, we found that VPA animals presented a significantly lower prevalence of patterns type 1 and 4 than the control group (Fig. 6*K*; K–W, *X*^2^ = 17.56, DF = 5; *p* = 0.014). These patterns were characterized by sustained and sharp sensory stimulation-evoked increases in spiking activity, respectively (Fig. 6*J*). On the other hand, VPA animals also showed a higher prevalence of pattern type 3 (Fig. 6*K*), which is characterized by deep pauses induced by each stimulus of the train (Fig. 6*J*). Altogether, these data confirm that S1 sensory-evoked responses were dominated by an inhibitory component in VPA animals.

The previous data suggest that, as a population, cortical VPA networks may be representing sensory stimuli in a different way than control networks. To further scrutinize this possibility, we applied PCA to the sensory-evoked responses over the whole neuronal population and compared population trajectories between VPA and control animals (see Materials and Methods). First, when plotting the first two PCs against time, we found that population dynamics from the control group were organized as rotational trajectories that followed the same path started by each stimulus of the train (Fig. 6*L*, black trace). However, population trajectories from VPA animals followed a disorganized path clearly different from that of the control group (Fig. 6*L*, blue trace). To formally quantify differences between groups, first we calculated the confidence interval for each PC by generating surrogate spike trains by randomly shifting the spike times in a range of ±5 s and plotting them against each component from the control and VPA groups (Fig. 6*M–O*, gray traces). In the control group, the three components presented an oscillatory shape with five cycles starting and corresponding to each stimulus of the train (Fig. 6*M–O*, black dots and trajectories); these cycles were well differentiated from the surrogate trajectories. On the contrary, VPA trajectories (Fig. 6*M–O*, blue dots and trajectories) were clearly oscillatory for PC1 but to a lesser extent for PC2 and PC3 (the latter was completely embedded in the surrogate trajectories). Then, we calculated the average speed and amplitude of each component for the three groups (surrogate, control, and VPA) and performed statistical comparisons. We found no differences in speed or amplitude for PC1, but significant differences between control and VPA animals for PC2 and PC3, being the former significantly faster and wider (Fig. 6*P*; K–W, *X*^2^ = 248.3, DF = 5; *p* < 0.001). On the other hand, the amplitude of the first component was significantly higher, while the amplitudes of components two and three were significantly lower in VPA animals (Fig. 6*Q*; K–W, *X*^2^ = 337.1, DF = 5; *p* < 0.001). In both groups, PC1 accounted for ∼25% of the population variability, and PC1 trajectories were characterized by a slow oscillatory component triggered by each stimulus of the train, suggesting that this component is associated with the large drops in activity triggered by each stimulus of the train (Fig. 6*C*). Hence, changes in the amplitude of PC1 reflect shifts in the general architecture of sensory-evoked responses. Moreover, the higher amplitudes observed in the VPA group are consistent with the previous quantifications, indicating that sensory-evoked activity is dominated by a decrease in activity in the S1 of VPA animals.

### Atypical somatosensory stimulus train-evoked neuronal activity in the DLSt of VPA-exposed animals

**Figure 7.**
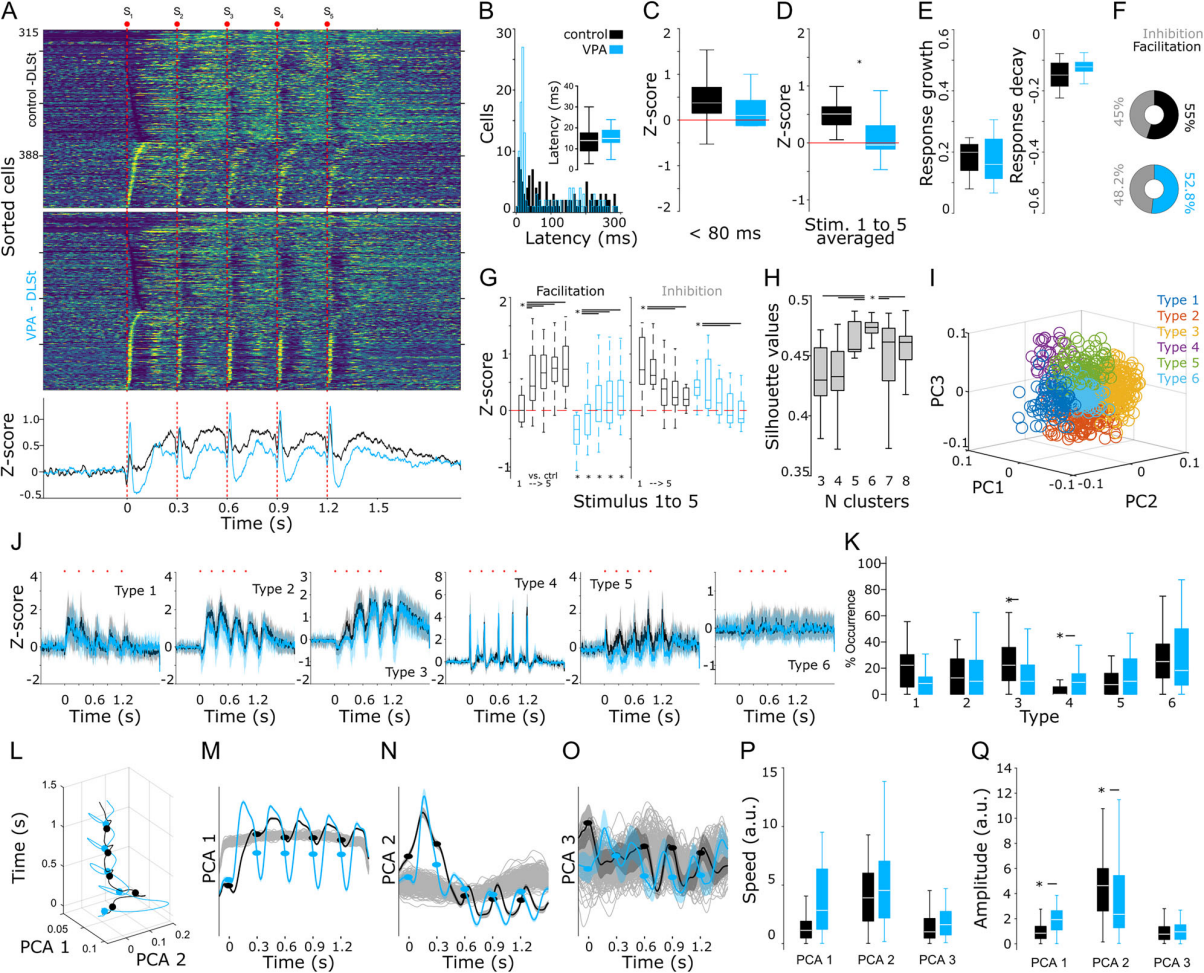
DLSt population dynamics in control and VPA animals. ***A***, Averaged firing rates for cells recorded in the DLSt of control (top) and VPA (bottom) animals expressed as *z*-score (range value, −1 to 1.5) and sorted according to the time they reached the highest (bottom to top) or lowest (top to bottom) activity after somesthetic stimulation (indicated by red dots and lines). Bottom traces represent averaged histograms of the population responses. ***B***, Response latencies to the first stimulus of the train; latencies under 50 ms are compared in the inset panel. For the entire figure, control and VPA groups are presented in black and blue, respectively. ***C***, ***D***, Amplitude of the general population responses for a 200 ms window starting 80 ms after the onset of the stimuli and for the joint five stimuli of the trains (left, S1–S5) or for each stimulus of the train. ***E***, Linear growth (left panel) or decay (right panel) rates for striatal train responses. ***F***, Percentage of neurons classified as facilitating or (solid color) or depressing (gray) based on their linear growth (or decay) adaptation profile to the progression of the stimulation train. ***G***, Comparison of the response amplitude for the long-latency increase component the sensory-evoked responses. Top asterisk and lines indicate intra-group statistical differences between the first stimulus of the train (asterisk) and the corresponding subsequent ones (joined by lines). ***H***, Silhouette values for 1,000 iterations in 3–8 *k*-means projections from the PCA on the peri-event histograms of the sensory-evoked spiking activity. ***I***, Best PC projection corresponding to six clusters (color coded). ***J***, Averaged sensory-evoked responses for cells classified as part of specific sensory-evoked patterns in control and VPA animals. Solid lines and shaded areas represent the median and the 25th and 75th percentiles, respectively. ***K***, Percentages of cells belonging to the different classified patterns in ***J***. ***L***, Low-dimensional PCA-3D projection of the population activity in S1 evoked by 50 stimulation trains for control, and VPA recordings. PC-2D projections for PC 1–3 (***M–O***) versus time. Gray lines represent 1,000 surrogate PCA projections obtained from shuffling spike trains. Comparison of the trajectory speed (***P***) and amplitude (***Q***) for each PC. For corresponding panels, boxplots indicate median and 75th and 25th percentiles. Statistical differences are indicated by asterisks and lines joining specific comparisons and obtained with K–W (as indicated in the main text) and the Bonferroni’s post hoc test (*p < 0.05; ***p < 0.001).

After analyzing S1 sensory-evoked activity, we proceeded to explore striatal responses to the same stimulation protocol. Consistent with previous reports (Hidalgo-Balbuena et al., 2019; Peña-Rangel et al., 2021), the averaged population responses of the DLSt in control animals displayed a transient increase in activity after the first stimulation pulse, followed by attenuated responses to the subsequent stimuli of the train (Fig. 7*A*). Neurons that presented short-latency responses to the first stimulus of the train (<30 ms) exhibited no significant differences among experimental conditions (Fig. 7*B*; Wilcoxon test, *p* = 0.121). Next, we analyzed the amplitude of the evoked responses. We divided the data considering the five-stimulus average amplitude of short-latency (<80 ms) and long-latency responses (200 ms windows starting 80 ms after the onset of each stimulus of the train). According to our findings, the short-latency responses did not differ significantly among groups (Fig. 7*C*; Wilcoxon test, *p* = 0.56). However, when analyzing the long-latency responses, we found that the VPA group presented significantly smaller amplitudes than the control group (Fig. 7*D*; Wilcoxon test, *p* = 0.04). As for S1 data, we identified neurons with facilitated or depressed responses by calculating the linear growth (or decay) rate of their response amplitudes to the progression of the somatosensory stimulation train. Striatal neurons in both groups presented similar growth and decay rates (Fig. 7*E*; growth: Wilcoxon test, *p* = 0.68; decay: Wilcoxon test, *p* = 0.87), with similar prevalences in their populations (Fig. 7*F*). We also calculated the amplitude of the responses to each stimulus of the train for the facilitating and depressing classified units. Similar to our findings in S1, we identified clear facilitating and depressing dynamics in both groups, as indicated by the significant differences between the amplitude of the first stimulus of the train and the following ones (Fig. 7*G*, top asterisk and lines; facilitating units K–W, *X*^2^ = 38.39, DF = 9; *p* < 0.001; inhibiting units, K–W, *X*^2^ = 27.44, DF = 9; *p* < 0.01). However, when we analyzed potential differences between the groups, we found that for the facilitating neurons, all train stimuli in control animals were significantly higher than those in the VPA group (Fig. 7*G*). On the other hand, for the depressing neurons, the VPA animals displayed significantly lower amplitudes than the control group for all stimuli of the train (Fig. 7*G*). These results indicate that VPA animals presented diminished responses to somatosensory stimulation.

We next applied our pattern classification method and found that, in the DLSt, the projection with the significantly highest silhouette values corresponded to six clusters (Fig. 7*H*,*I*; K–W, *X*^2^ = 3,159.2, DF = 5; *p* < 0.001) with clearly different shapes in response to somatosensory stimulation. These clusters were identified in the three experimental groups (Fig. 7*J*). When analyzing the pervasiveness of each pattern (K–W, *X*^2^ = 27.94, DF = 11; *p* = 0.003), we found that patterns 3 and 4 were significantly under- and over-represented in the VPA animals, respectively (Fig. 7*K*). On the other hand, patterns 1 and 2 were under-represented and pattern 3 was over-represented in PV + Cre-Casp animals when compared with the control group (Fig. 7*K*). Pattern type 4, overexpressed in VPA animals, consisted of sharp, short-latency responses that increased with each stimulus of the train, which would explain the short-latency facilitation observed in the average population responses (Fig. 7*A*, bottom panel). Next, we analyzed the population dynamics with PCA and discovered that, in control conditions, contrary to what was observed in S1, DLSt presented more discrete oscillatory dynamics in PC1 and PC2, with PC3 almost completely embedded in the surrogate data (Fig. 7*L–O*). In contrast, VPA striatal dynamics had wide oscillatory components in PC1 and PC2, which was reflected in significantly faster speeds (Fig. 7*P*; K–W, *X*^2^ = 340.3, DF = 5, *p* < 0.001) and wider amplitudes (Fig. 7*Q*; K–W, *X*^2^ = 543.5, DF = 5, *p* < 0.001) when compared with the control group.

**Figure 8.**
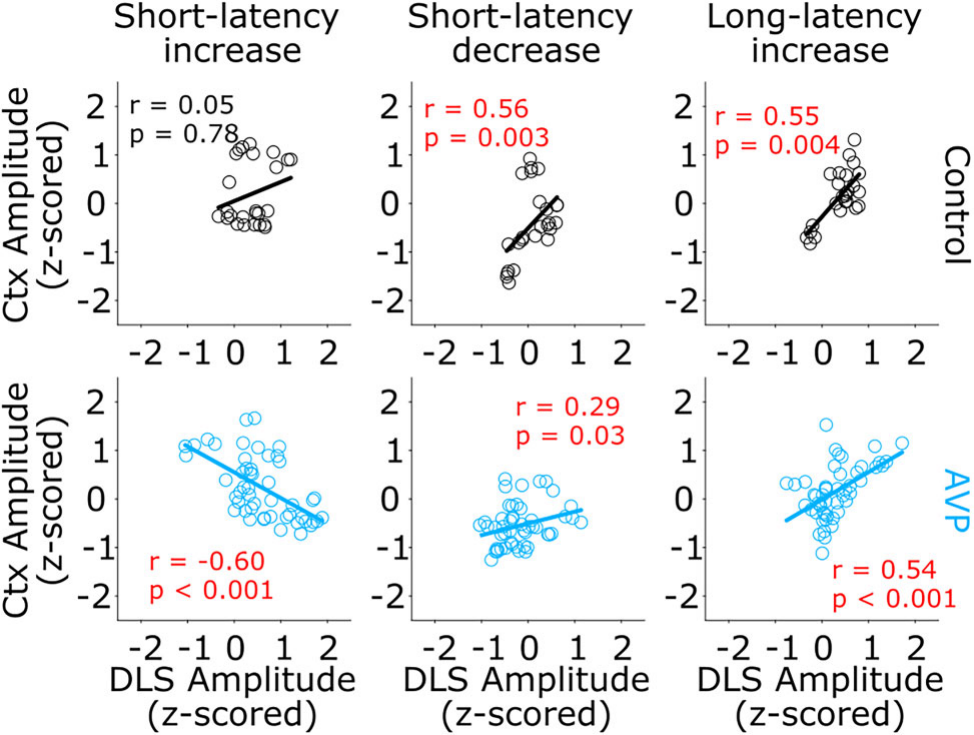
S1-DLSt correlations. Spearman correlation plots between cortical S1 (*Y* axis) and DLSt (*X* axis) for the amplitude of the short-latency increase (left column) and decrease activity (middle column) and the amplitude of the long-latency increase component (right column) of the sensory-evoked responses in the control (top row) and VPA animals (bottom row). Correlation and probability values are displayed for each comparison (significant comparisons are highlighted in red).

Our electrophysiological observations indicate that striatal activity in the VPA animals presented slightly higher baseline firing frequencies than those of control animals, accompanied by evoked responses with lower amplitudes in the increased activity component. This resulted in average population dynamics dominated by strong oscillatory pauses triggered by each stimulus of the train. But an important question is: what relationship is there between striatal and cortical dynamics? While our cortical and striatal recordings were not performed simultaneously, they were performed in the same session and animals, allowing us to calculate corticostriatal correlations of sensory-evoked data. We compared the amplitude of the short-latency increase (Fig. 8, left column) and decrease activity (Fig. 8, middle column) and the amplitude of the long-latency increase component (Fig. 8, right column) of the sensory-evoked responses in the control (Fig. 8, top row) and VPA animals (Fig. 8, bottom row). In control animals, the short-latency decrease and long-latency rebound presented significant positive correlations between the S1 and DLSt. This relationship was maintained in VPA animals. However, contrary to what happened in the control group, the amplitude of the short-latency increase in VPA animals exhibited a significant negative correlation. These results suggest that, in addition to the intrastructural changes, corticostriatal communications may also be altered in VPA animals.

**Figure 9.**
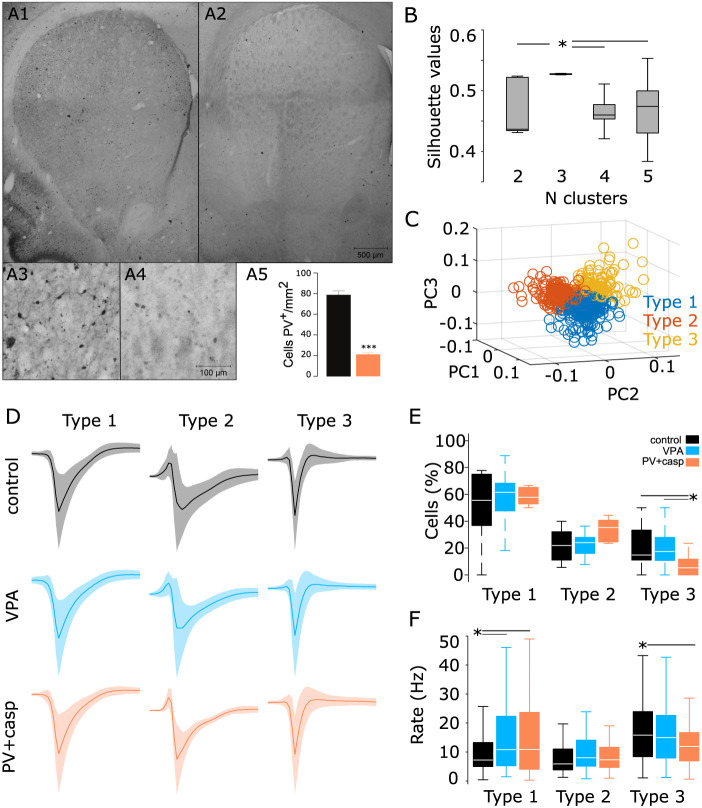
Baseline activity in subpopulations of striatal neurons. Representative microphotographs (***A1–A4***) and quantifications (***A5***; control 78.64 ± 3.831 and PV + Cree-Casp 21.16 ± 1.657) of the NSt from control (***A1***, ***A3***) and PV + Cre-Casp (***A2***, ***A4***) animals showing the distribution of PV labeling; ***p < 0.001. ***B***, Silhouette values for 1,000 iterations in 5–5 *k*-means projections from the PCA on the spike-wave shapes for striatal neurons. ***C***, Best PC projection corresponding to three clusters (color coded). ***D***, Average spike waves from all cells classified as belonging to specific subpopulations in control (black code for the entire figure), VPA (blue code for the entire figure), and PV + Cre-Casp animals (orange code for the entire figure) recorded in the DLSt. ***E***, Percentage of cells classified as belonging to each subpopulation. ***F***, Firing rates for all cells recorded in the DLSt belonging to each subpopulation.

On the other hand, our anatomical data suggest that striatal dynamics may be affected by the changes in interneuron proportions or functionality (Figs. 2, 3). To further scrutinize in the mechanism of these general population changes, we aimed to classify striatal neurons based on their spike shapes. However, studies using immunoreactivity methods have reported that PV + neuron hypoactivity may be related to a decrease in PV protein cell levels (Filice et al., 2016; Donato et al., 2013). Hence, we complemented our study by also investigating whether PV-specific depletion could have any electrophysiological effects in this region. To do so, we used a genetically targeted lesioning approach with a new group of animals (*n* = 4). We injected the adeno-associated virus pAAV-flex-taCasp3-TEVp into the DLSt to induce Caspase 3 expression and apoptosis in PV^+^ neurons from transgenic rats, where Cre-recombinase is expressed under the PV promoter (PV + Cre-Casp; Fig. 9*A*). Then, we applied the same PCA/silhouette-based method of classification used to identify sensory-evoked response patterns, but in this case, we classified spike-wave shapes and estimated the absolute proportion of putative MSNs, putative fast-spiking interneurons, and others (Peña-Rangel et al., 2021). By applying this method, we found that in this striatal dataset from the three experimental groups, spike shapes could be grouped into a three-cluster projection (Fig. 9*B–D*; K–W, *X*^2^ = 859.8, DF = 3, *p* < 0.001). Most cells recorded corresponded to a wider shape and the lowest firing rates, consistent with MSNs (Fig. 9*D*,*E*, control 55.5%; VPA 61.5%; PV + Cre-Casp 52.9%; K–W *X*^2^ = 37.26, DF = 8, *p* < 0.001). However, this type of neurons presented significantly higher firing frequencies in VPA and PV + Cre-Casp animals than in control animals (Fig. 9*E*; K–W, *X*^2^ = 21.16, DF = 8, *p* = 0.006). We did not find significant differences in the proportion or baseline firing rates of the type 2 units. However, type 3 units, whose firing rates and spike-wave shapes are consistent with fast-spiking interneurons, presented significantly lower prevalence (Fig. 9*E*) and significantly lower firing rates (Fig. 9*F*) for PV + Cre-Casp animals.

**Figure 10.**
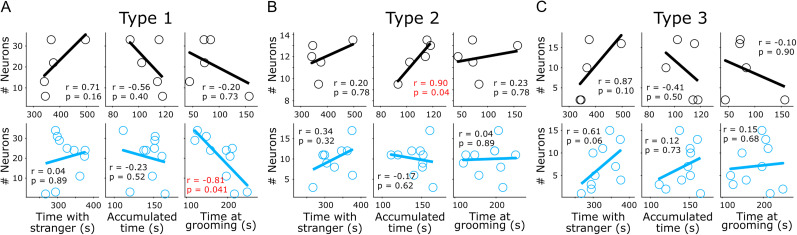
Correlations between striatal subpopulations and behavioral variables. Spearman correlation plots between the number of Type 1 (***A***), Type 2 (***B***), and Type 3 (***C***) neurons and different behavioral variables used to confirm the VPA model for the control (black, top row) and VPA animals (blue, bottom row). Correlation and probability values are displayed for each comparison (significant comparisons highlighted in red).

The previous results suggest that even when the histological data show a decrease in the PV^+^ and ChAT^+^ signals (Figs. 2, 3), the effects observed in the striatal population dynamics of VPA animals are not explained by the specific loss of PV^+^ interneurons. Instead, they are most likely explained by a general striatal imbalance. But would these altered neural dynamics explain the behavioral effects observed in VPA subjects? To answer this question, we tried to capitalize on the fact that most VPA-treated animals (*n* = 10) and some control animals (*n* = 5) used for behavioral confirmation of the VPA model were also recorded under anesthetized conditions after behavioral assessments. Here, for each animal, we intended to correlate the number of neurons classified as putative MSNs (type 1), others (type 2), or putative fast-spiking interneurons (type 3) with their corresponding behavioral values (total time of interaction with the stranger, Fig. 1*A*; accumulated time fixed on the light, Fig. 1*C*; and time spent on self-grooming, Fig. 1*B*). We found that VPA animals presented a robust and significant negative correlation between the total number of neurons classified as putative MSNs and the grooming time in the open field; that is, the more putative the MSNs, the less time spent on grooming. On the other hand, in the control group, neurons classified as “others” presented a robust positive correlation with fixation time in the hole-board test, and this correlation was lost in the VPA animals (Fig. 10*B*). Finally, neurons classified as putative fast-spiking interneurons displayed no significant correlations in either group. While these experiments are substantially limited by the lack of simultaneous electrophysiological and behavioral observations, it suggests that the general imbalance in neural dynamics identified in the NSt may partially explain some of the behavioral features observed in the VPA model.

## Discussion

In the present work, we used animals prenatally exposed to VPA as an ASD model. Consistent with previous reports, VPA animals showed deficits in social behavior, difficulty in reorienting their attention to a particular sensory stimulus, and increased stereotyped behavior, indicating a reliable recapitulation of the ASD model. These behavioral alterations were accompanied by a decrease in both ChAT^+^ and PV^+^ signals in the NSt, with a more significant reduction observed at the DLSt level. A limitation of this observation is that, while a decrease in ChAT^+^ and PV^+^ signals could be interpreted as a decrease in total number of neurons, it is possible that these reduced signals could make it more difficult to identify interneurons without a real decrease in the total number of units. Nevertheless, a decrease in these signals together with a consistent behavioral outcome suggest that information processing in the DLSt may be altered in VPA animals. To functionally study this possibility, we performed high-density electrophysiological recordings in anesthetized animals. In the control animals, S1 cortical population dynamics presented clear rotational trajectories (Fig. 6*L–O*), while striatal dynamics presented smaller amplitudes in the rotational component (Fig. 7*L–O*). Being a primary somatosensory region, the high amplitude in the rotational trajectories observed in S1 is explained by the robustness in representing each stimulus of the train, with virtually all neurons expressing one of the four oscillatory response patterns depicted in Figure 6*J*,*K*. On the other hand, the lower amplitude in DLS is explained by the fact that in this region an important percentage on neurons (∼30%) did not express a clear response to the somatosensory stimulation (Pattern type 6; Fig. 7*J*,*K*). When comparing control and VPA-exposed animals, we found that the later ones presented altered neuronal dynamics in both the S1 and DLSt in response to the cutaneous somatosensory stimulation, with the DLSt area having a more exacerbated response, resembling the cortical rotational population trajectories (Figs. 6*L–O*, 7*L–O*). To determine whether these differences were explained by alterations in specific interneuron subpopulations, we performed targeted elimination of striatal PV^+^ neurons and observed that these animals also exhibited strong alterations in sensory-evoked neural dynamics but only partially recapitulated the VPA effects, indicating a higher complexity in the VPA phenotype. Furthermore, altered neural dynamics characterized by fastest and wider population trajectories were observed in the DLSt of VPA animals. Interestingly, these dynamics were similar to those observed in the S1 of control animals, suggesting the loss of a hypothetical filtering function in the DLSt of VPA animals. In this study, we focused the histological quantifications on interneuron populations. This could be a limitation, considering that MSNs are the most prevalent cell type in the DLSt. However, our electrophysiological quantifications included all possible cells recorded. Moreover, when subdividing the populations based on the spike waveform analysis, we found that in VPA and PV + Cre-Casp animals, units with spike shapes that were consistent with MSNs significantly increased their firing rates compared with control animals. This finding indicates that the VPA model affects the general dynamic of the NSt. A controversial observation is that, in the VPA animals, we did not find changes in the gamma frequency or in any other oscillatory frequency. However, this could be explained by the fact that we did not measure gamma directly in the local field potential but in the oscillatory component of the autocorrelogram of individual neurons. On the other hand, we did not study the potential effects of this model in striatal non-neural populations, but other studies have researched these effects in other brain regions using the VPA exposure model and have shown that the model induces alterations in the glial system. These alterations can lead to various developmental, anatomical, and physiological effects and have been attributed to disturbances in various glial elements, such as S100b, GABAA-ρ3, and glial fibrillary acidic protein in astrocytes, Olig2 in oligodendrocytes, and CD11B and Iba1 in microglia (Codagnone et al., 2015; Bronzuoli et al., 2018; Gąssowska-Dobrowolska et al., 2020; Traetta et al., 2021; Saavedra-Bonilla et al., 2024). These findings suggest that this model may also affect the striatal glial system, but more research is needed to confirm this hypothesis.

### ASD model

As demonstrated in previous studies validating different models of ASD (Rodier et al., 1997; Schneider and Przewłocki, 2005; K. C. Kim et al., 2011; Schiavi et al., 2019; Elnahas et al., 2020), we observed an important reduction in the social behavior of VPA animals, which displayed fewer contacts and interactions with a novel rat (K. C. Kim et al., 2011; Foley et al., 2014). Furthermore, animals also exhibited aberrantly long periods of attention engagement to a visual stimulus (Fig. 1*C*). These behaviors are typically observed in ASD patients, with reduced social interaction and communication, resulting in poor social performance in their environment (Franchini et al., 2017; Supekar et al., 2018). Likewise, these patients also display hyper- or hypo-responses to sensory stimuli (Fontes-Dutra et al., 2018; Siemann et al., 2020), as well as low attentional levels to the general environment while being hyper-engaged to a specific stimulus (Orekhova and Stroganova, 2014). Finally, VPA animals also displayed an increase in stereotypical behaviors, as exemplified by the exacerbated frequency and duration of grooming (Hartgraves and Randall, 1986; Wu et al., 2019; Li et al., 2024). This finding is consistent with previous reports (Langen et al., 2014; J. W. Kim et al., 2017; Hirsch et al., 2018). Altogether, our model successfully mimics important features of autistic behavior.

### Decrease in ChAT^+^ and PV^+^ cells in the NSt

Although interneurons are a small percentage of cells in the NSt (∼5%), they play an important role in the processing of different synaptic inputs reaching the NSt (Tepper and Bolam, 2004; Tepper et al., 2010, 2018) and in the control of synaptic output and the inhibition–excitation balance of neuronal circuits. In this sense, ChAT^+^ and PV^+^ cells are two central elements for the proper functioning of the NSt. ChAT^+^ interneurons provide the main source of acetylcholine (Ach) to the NSt (Zhou et al., 2002; Shen et al., 2005; Abudukeyoumu et al., 2019), while PV^+^ interneurons control NSt synaptic output by inhibiting MSNs (Tepper et al., 2018).

Diverse studies show that a deregulation in the cholinergic system can generate differences in social and cognitive behavior, increasing stereotypic patterns in patients with ASD and Tourette syndrome, as well as in animal models (Kataoka et al., 2010; Oginsky et al., 2014; Rapanelli et al., 2017b). In this sense, our results showed a significant reduction of ChAT^+^ levels in the NSt of VPA animals (Fig. 2). This reduction suggests a weak cholinergic modulation in the NSt, which would partly explain the increased grooming and social deficits in our VPA animals (Cubo et al., 2008; Karvat and Kimchi, 2014). Interestingly, the spatial configuration of the ChAT^+^ distribution was altered along the NSt, with the DLSt being the most affected, losing the rostral-caudal and medial-lateral gradient observed in control animals (Fig. 2; Meredith et al., 1989; Matamales et al., 2016a). Similar to ChAT levels, PV levels were significantly reduced (Fig. 3). These results are in agreement with previous reports using genetic and pharmacological models of ASD (Peñagarikano et al., 2011; Filice et al., 2016; Lauber et al., 2016). Furthermore, Orduz and colleagues (Orduz et al., 2013) found that low PV expression led to electrophysiological alterations in fast-spiking neurons and, therefore, to a deficit in the modulation of afferents reaching the NSt, particularly the MSN (Monterio et al., 2018). Likewise, Rapanelli and collaborators (2017) showed that a joint depletion of ChAT^+^ and PV^+^ cells in the NSt emulates the main characteristics of ASD. However, previous studies have reported that PV^+^ neuron hypoactivity may be related to a decrease in PV protein expression and may be related with ASD (Filice et al., 2016; Donato et al., 2013; Contractor et al., 2021). In our data, the decreased levels in PV^+^ (Fig. 3) were not accompanied by a decrease in the prevalence of neurons with sharp spike waves shapes, typically associated to FSI PV^+^ interneurons (Fig. 9), opening the possibility that our data could be better explained by a decreased in the levels of PV protein and not an absolute decrease in the number pf PV^+^ neurons. This possibility was explored by experimentally depleting PV^+^ neurons in a transgenic rat model (Fig. 9). This manipulation induced decrease in both, the levels of PV^+^ neurons and the prevalence of neurons expressing sharp spike waves. This data suggests that in the VPA model an imbalance in the basal ganglia interneuron functionality is related to atypical somatosensory processing. In this context, striatal interneurons exert considerable control over MSNs and help in the processing of cortical and thalamic afferents and regulation of their synaptic output (Tepper and Bolam, 2004; Tepper et al., 2010, 2018; Marín, 2012; Kepecs and Fishell, 2014; Poppi et al., 2021). Our data show a heterogeneous decrease in ChAT^+^ and PV levels in the NSt of VPA animals. This reduction was accompanied by changes in the cortical and striatal network dynamics. Notably, in the NSt, VPA animals expressed a modest increase in baseline firing rates (Fig. 4*D*) that appeared to be associated with the activity of putative MSNs (Fig. 9) and, most importantly, different somatosensory-evoked responses to each of the sensory stimuli of the train (Fig. 7). When compared with the control animals, VPA animals presented different adaptation profiles, with lower amplitudes in the “rebound” long-latency increase component of the response. These changes produced a net imbalance in the responses, which were dominated by stronger pauses in activity, as seen in the average population response (Fig. 7*A*, bottom panel) and PC1 amplitude (Fig. 7*A*,*Q*). A possible explanation for these different responses would implicate an imbalance in ChAT^+^ and PV levels within the NSt, affecting their ability to adequately filter and process synaptic commands from the S1. It is important to highlight that the selective ablation of PV^+^ cells did not fully recapitulate the VPA phenotype. This finding indicates that the VPA model does not affect a particular neural population but causes a generalized imbalance. In this context, our data suggest that the interneuron imbalance would impact on sensory-evoked network activity in the dorsolateral striatum. Since striatal interneurons will not project outside the striatum, the behavioral outcome associated to these effects must implicate the main striatal neurons, MSNs, and their canonical connectivity within the BG (i.e., direct and indirect pathways). Here, our correlation analysis between the proportion of subpopulation of striatal neurons and behavioral parameters (Fig. 10) suggests a relationship between the number of putative MSNs (Type 1) and grooming behavior. However, while in our dataset we did not evaluate the levels of MSNs, diminishing the scope of our interpretations, these data confirm that linking neural dynamics and behavioral outcomes associated to the VPA model would require a wider approach where all the elements of striatal microcircuit are taken into consideration. To understand this imbalance, further research is needed, as well as experimental designs specifically targeting different elements of the striatal and cortical circuits. These data are in agreement with other studies showing that developmental differences or interneurons would lead to a series of electrophysiological and behavioral differences in sensory processing (Rapanelli et al., 2017b; Filice et al., 2020; Q. Chen et al., 2020).

The decrease in ChAT^+^ cells within the NSt would lead to a decrease in ACh concentration in VPA animals, which is consistent with the study by Karvat and Kimchi (2014), where the authors used an acetylcholinesterase inhibitor directly infused in the NSt and demonstrated cognitive and social interaction improvements in a mouse model of ASD. According to previous literature, a decrease in striatal ACh levels would lead to impaired modulation of corticostriatal afferents through type 2 muscarinic acetylcholine receptors that negatively control cortical glutamate release to the NSt (Calabresi et al., 2000). Likewise, feedback and feedforward inhibition would also be affected, since these processes have been linked to the activation of nicotinic receptors expressed in the GABAergic interneurons of the NSt (Koós and Tepper, 2002; English et al., 2011; Faust et al., 2016; Assous et al., 2017). Furthermore, it has recently been proposed that cholinergic interneurons (CINs) can indirectly modulate cortical MSN communication through PV^+^ interneurons (Matityahu et al., 2022). This effect that can be directly altered by CIN distribution across the entire NSt (Fig. 2; Dorst et al., 2020). Moreover, our data also indicate that CIN modulation directly over MSNs would also be affected, since MSNs express muscarinic receptors at pre- and postsynaptic levels (Goldberg et al., 2012; Assous, 2021). While further experiments would be necessary to causally link a decrease in ChAT^+^ cells (and therefore in Ach) within the NSt with the atypical sensory-evoked responses observed in our data, the previous literature will favor this interpretation. Additionally, the observed decrease in PV^+^ cells would reinforce the higher baseline frequencies and different sensory-evoked responses at the DLSt level in VPA animals due to a deficit in the inhibitory elements of the striatal circuit (Marín, 2012; Tepper et al., 2010, 2018). A decrease in PV^+^ interneurons may also impact on general striatal processing, especially in the DLSt, since it has been proposed that these interneurons are organized and communicate through gap junctions (Koós and Tepper, 1999). Altogether, our results may help to understand the exacerbated behaviors that ASD patients express in response to sensory stimulation, which can vary in intensity and frequency and that on many occasions may even generate crises (Buckley and Holmes, 2016).

## Synthesis

Reviewing Editor: David Franklin, Technische Universitat Munchen Decisions are customarily a result of the Reviewing Editor and the peer reviewers coming together and discussing their recommendations until a consensus is reached. When revisions are invited, a fact-based synthesis statement explaining their decision and outlining what is needed to prepare a revision will be listed below. The following reviewer(s) agreed to reveal their identity: Patricia Monteiro. Note: If this manuscript was transferred from JNeurosci and a decision was made to accept the manuscript without peer review, a brief statement to this effect will instead be what is listed below.

Your manuscript has now been reviewed by two expert reviewers. Unfortunately, we were unable to obtain the two previous reviewers of your manuscript. After coming together to discuss the manuscript we believe that the manuscript is potentially suitable for eNeuro. However, we all share concerns with the current version of the manuscript. In particular we have concerns that a control condition is missing for the PV+Cre-Casp condition, as well as issues with the current statistical analysis and other methodological concerns. Nevertheless, we appreciate the contributions of the work and would be willing to consider a revision provided the concerns can be addressed. Please consider the comments provided by the reviewers and prepare a revision that takes these into account, along with a point-by-point response.

Specific comments from the reviewers.

Reviewer 1.

The authors use a rat model for the study of autism spectrum disorder, in which prenatal valproic acid exposure leads to behavioral alterations that may relate to ASD-like phenotypes and changes in electrophysiological properties of neurons in somatosensory cortex and striatum. The authors report some interesting findings but there are methodological issues that temper enthusiasm. I have made suggestions below.

1. PV+Cre-Casp experiment: my reading of the manuscript suggests that no control group was used for this experiment, and that the post hoc addition of this cohort was statistically compared to the control animals from the VPA experiments. This is unacceptable for several reasons. Not only are cohort/timing fully confounded, but the conditions aren't controlled for - the control and VPA animals are offspring that underwent a parallel manipulation, prenatally. The PV+Cre-Casp rats underwent intracranial surgery, as adults. I also don't see anything in the manuscript about a histological verification of the success of the viral surgeries. Finally, the PV+Cre-Casp rats were on a Long-Evans background, whereas the control/VPA rats were Sprague-Dawley. As such, I don't think any of the comparisons made between PV+Cre-Casp rats and control or VPA rats are sound - what if the disparities between the VPA model and the Casp rats are actually attributable to genetic background, intracranial surgery, prenatal challenge, or cohort? While this issue could be mitigated by adding a control surgery group (e.g., with a control virus infused) with the same transgenic rat line, there would still be the issue of cohort confounding unless additional rats are also added to the PV+Cre-Casp group in the subsequent data collection to mitigate this effect. Therefore, this data should probably just be removed from the manuscript or the experiment redone with a concurrent control group.

2. Statistics: as mentioned by a prior reviewer, all statistics should be done with the individual animals as the experimental units, not cells (either average the cells within subject or make a hierarchical statistical model). I believe the authors modified panel 4D in response to that comment, but no other statistics or figures seem to be updated accordingly. That panel illustrates the main issue with pooling cells across animals - it can lead to type 1 error due to lack of accounting for interdependence of data points (e.g., within-subject effects). This issue is very nicely explained in a recent Neuron primer by Yu et al., 2022 ("Beyond t test and ANOVA"). In the individual cell result for 4D, they report increased firing in both S1 and DLSt, whereas this only reaches significance in the DLSt when done by animal, so the S1 significance is lost. Yet, the text reports "a greater excitability in the cortical and striatal networks of VPA animals (Fig. 4D)." All the pooled statistics with cells and depths should be redone to account for animal throughout the manuscript, including the correlations. Test statistics and degrees of freedom would be helpful to report in addition to p-values, which the authors did for some of the recently added results text but not throughout much of the original text. Also, correlations with few observations (approximately <30) are more appropriately performed with a Spearman rank test than a Pearson, which is more sensitive to outliers particularly with small sample sizes.

3. Clarity on VPA model: It is difficult to discern the novelty and/or consistency with the literature of the findings because the literature summaries tend to generalize rather than reporting when the specific VPA model in rats has been used. The authors frequently refer to ASD rodent models generally instead, which is also useful, but doesn't contextualize the specific niche of these findings. VPA is not mentioned within the introduction. When contextualizing the behavioral results, they are compared generally to ASD models without specifying VPA or species. The same is true when introducing the Figure 2 results on ChAT+ and PV+ cell counts.

4. Behavior: the authors provide anxiety in ASD as rationale for performing the open field test. However, they do not measure any classical anxiety-like phenotypes in this assay. The open field test is designed to induce thigmotaxis, which can be estimated using time spent in the center of the arena or %distance traveled in the center. If the authors have this information, they should add it, and if they don't, then the authors shouldn't say they are measuring anxiety-like behavior. As a prior reviewer mentioned, the social interaction test used here doesn't include an object control or a familiar mouse control, so the construct being captured is ambiguous. The authors added a section at the end of the behavioral results to mention this caveat, but it conflicts with an earlier statement claiming that there couldn't be confounds due to exploration in this assay. The authors state that grooming is a "commonly accepted indicator of increased stereotyped behavior." This should be backed up with citations. The final interpretation of the hole board test is bordering on anthropomorphizing, so I think it would be best to rephrase the statement regarding rats being hooked to the light stimulus.

5. Additional detail on animal numbers: please add the number of pregnant dams used, how many of the behavioral animals were from how many different litters, specify that the electrophysiology experiments selected only one animal from each litter (from what I read in the prior review response), and when reporting the number of neurons recorded from each group, break that down by animal. Also, pg. 20 says 12 control rats were used for the electrophysiology but doesn't specify the number of VPA rats, and then pg. 38 says "most VPA-treated animals (n = 10) and some control animals (n = 5) used for behavioral confirmation of the VPA model were also recorded under anesthetized conditions..." If only 5 of the control animals used for behavior were also used for electrophysiology, but 12 control rats were used for electrophysiology, does that mean there is a mix of animals that did and did not undergo behavioral testing that then went on to undergo electrophysiology?

6. Electrophysiology results details: perhaps this is only because I'm not an electrophysiology expert, but I found these results, particularly for figures 6 and 7, very dense with methodological detail that made it hard to pull out the functional meaning. I would consider moving some of the information about the analyses used to the methods, which might make it easier to find in the results what is functionally being represented by the various measures reported (e.g. what the silhouettes, clusters, patterns, types signify).

7. As a prior reviewer pointed out, it's unexpected that the counts for FSIs aren't reduced in the VPA group (Fig. 9). I think this should be mentioned in the discussion in the new caveat section where the authors discuss the idea of reduction in expression of PV vs number of PV+ neurons.

8. I don't think the authors address Fig. 10 in the discussion. I think it would be worth discussing that the putative FSIs don't correlate with these behaviors but that the MSNs do and contextualizing that in the literature.

Minor:

1. In the methods, explicitly state the rationale for exclusive use of male animals

2. Literature on VPA models &glia in striatum: there is a very recently published paper, see Saavedra-Bonilla et al., Neurochem Res, 2024.

3. Statement at the end of behavior section on statistics is repeated in the statistics section. I would just keep it in the statistics section and delete from the behavior section.

4. The authors oscillate between including and not including the "+" for "PV" (vs "PV+") and ChAT (vs "ChAT") inconsistently. The "+" should be there for when referring to it as a marker, such as a "PV+ cell", whereas when referring to the protein itself, the + should not be included.

5. In several places in text and figures the authors write "AVP" instead of "VPA"

6. I think Fig 6G is meant to have an asterisk to indicate significance

7. On page 33 line 681 I think it is a typo when the authors say "...contrary to S1 animals, control animals..."

8. Line 770: typo "bee" for "be"

Reviewer 2.

This manuscript investigates striatal interneurons in a rat model of ASD induced by prenatal exposure to valproic acid (VPA). The authors validate the animal model through relevant behavioral assays, quantify interneuron numbers using immunohistochemistry, and conduct electrophysiological recordings in both striatal and cortical regions. The paper has undergone extensive revision, with the authors incorporating a new set of experiments in which they selectively ablated PV neurons using a Cre/Caspase-based strategy. Additionally, the authors have refined some of their claims to better align with the data presented. I have only a few minor comments:

Figure 7L: The population dynamics in the control group appear to have lost the organized rotational trajectories that followed the same path initiated by each stimulus in the train, as previously observed in Figure 6L. The authors could address this discrepancy in their discussion.

Lines 652, 654, 658: The term "AVP group" should be corrected to "VPA group."

## Author Response

Reviewer 1.

The authors use a rat model for the study of autism spectrum disorder, in which prenatal valproic acid exposure leads to behavioral alterations that may relate to ASD-like phenotypes and changes in electrophysiological properties of neurons in somatosensory cortex and striatum. The authors report some interesting findings but there are methodological issues that temper enthusiasm. I have made suggestions below.

1. PV+Cre-Casp experiment: my reading of the manuscript suggests that no control group was used for this experiment, and that the post hoc addition of this cohort was statistically compared to the control animals from the VPA experiments. This is unacceptable for several reasons. Not only are cohort/timing fully confounded, but the conditions aren't controlled for - the control and VPA animals are offspring that underwent a parallel manipulation, prenatally. The PV+Cre-Casp rats underwent intracranial surgery, as adults. I also don't see anything in the manuscript about a histological verification of the success of the viral surgeries. Finally, the PV+Cre-Casp rats were on a Long-Evans background, whereas the control/VPA rats were Sprague-Dawley. As such, I don't think any of the comparisons made between PV+Cre-Casp rats and control or VPA rats are sound - what if the disparities between the VPA model and the Casp rats are actually attributable to genetic background, intracranial surgery, prenatal challenge, or cohort? While this issue could be mitigated by adding a control surgery group (e.g., with a control virus infused) with the same transgenic rat line, there would still be the issue of cohort confounding unless additional rats are also added to the PV+Cre-Casp group in the subsequent data collection to mitigate this effect. Therefore, this data should probably just be removed from the manuscript or the experiment redone with a concurrent control group.

R. Following the reviewer's advice, we removed the majority of quantifications for the PV+Cre-Casp group (originally depicted in Fig. 7). However, we have mantained PV+Cre-Casp group data for the basic spikewave shape analysis. In this case the PV+Cre-Casp data is only used as methodological confirmation four our clasification procedure.

2. Statistics: as mentioned by a prior reviewer, all statistics should be done with the individual animals as the experimental units, not cells (either average the cells within subject or make a hierarchical statistical model). I believe the authors modified panel 4D in response to that comment, but no other statistics or figures seem to be updated accordingly. That panel illustrates the main issue with pooling cells across animals - it can lead to type 1 error due to lack of accounting for interdependence of data points (e.g., within-subject effects). This issue is very nicely explained in a recent Neuron primer by Yu et al., 2022 ("Beyond t test and ANOVA"). In the individual cell result for 4D, they report increased firing in both S1 and DLSt, whereas this only reaches significance in the DLSt when done by animal, so the S1 significance is lost. Yet, the text reports "a greater excitability in the cortical and striatal networks of VPA animals (Fig. 4D)." All the pooled statistics with cells and depths should be redone to account for animal throughout the manuscript, including the correlations. Test statistics and degrees of freedom would be helpful to report in addition to p-values, which the authors did for some of the recently added results text but not throughout much of the original text. Also, correlations with few observations (approximately <30) are more appropriately performed with a Spearman rank test than a Pearson, which is more sensitive to outliers particularly with small sample sizes.

R. As suggested, we have now substituted the majority of the analyses grouping data by animal and not as individual neurons, except for panels Q and P in figures 6 and 7, since neural population analysis relays on the estimation of the variability of the whole population of neuorns. Following the reviewrs advice, we have also calculated and reported Spearman correlations for for Fig. 8 and 10.

3. Clarity on VPA model: It is difficult to discern the novelty and/or consistency with the literature of the findings because the literature summaries tend to generalize rather than reporting when the specific VPA model in rats has been used. The authors frequently refer to ASD rodent models generally instead, which is also useful, but doesn't contextualize the specific niche of these findings. VPA is not mentioned within the introduction. When contextualizing the behavioral results, they are compared generally to ASD models without specifying VPA or species. The same is true when introducing the Figure 2 results on ChAT+ and PV+ cell counts.

R.

We apologize for this ambiguity and thank the reviewer for this comment helping to improve the clarity of our results. We have now extended our description of the VPA model in the introduction and discussion sections. We have also improved descriptions including further details related to the species and ASD models and specified when the VPA was used.

4. Behavior: the authors provide anxiety in ASD as rationale for performing the open field test. However, they do not measure any classical anxiety-like phenotypes in this assay. The open field test is designed to induce thigmotaxis, which can be estimated using time spent in the center of the arena or %distance traveled in the center. If the authors have this information, they should add it, and if they don't, then the authors shouldn't say they are measuring anxiety-like behavior. As a prior reviewer mentioned, the social interaction test used here doesn't include an object control or a familiar mouse control, so the construct being captured is ambiguous. The authors added a section at the end of the behavioral results to mention this caveat, but it conflicts with an earlier statement claiming that there couldn't be confounds due to exploration in this assay. The authors state that grooming is a "commonly accepted indicator of increased stereotyped behavior." This should be backed up with citations. The final interpretation of the hole board test is bordering on anthropomorphizing, so I think it would be best to rephrase the statement regarding rats being hooked to the light stimulus.

R.

We agree with the reviewer's comment about the open-field test. Hence, we have removed the references to anxiety-like behavior, and we are only focusing on the results showing grooming behavior. We have also included the appropriate references where this behavior has been used as an indicator of stereotyped behavior (Hartgraves et al., 1986; Wu et al., 2019; Li et al, 2024). In the case of social interaction, for both groups, control and VPA animals, we preformed 5 minutes of habituation prior the 10 minutes social interaction test. On the other hand, we have now clarified that our results are limited to the interaction with a novel rat and not to social preference, as could also be measured by introducing a familiar vs. a novel animal (Kim et al, 2017). Finally, to avoid anthropomorphizing, we have now rephrased the referred statement as follows: "... suggesting that the VPA-exposed animals maintained a higher level of attention to the light stimulus" New references: • Hartgraves, S. L., &Randall, P. K. (1986). Dopamine agonist-induced stereotypic grooming and self-mutilation following striatal dopamine depletion. Psychopharmacology, 90(3), 358-363. https://doi.org/10.1007/BF00179191. • Wu, W. L., Cheng, S. J., Lin, S. H., Chuang, Y. C., Huang, E. Y., &Chen, C. C. (2019). The Effect of ASIC3 Knockout on Corticostriatal Circuit and Mouse Self-grooming Behavior. Frontiers in cellular neuroscience, 13, 86. https://doi.org/10.3389/fncel.2019.00086. • Li, G., Lu, C., Yin, M., Wang, P., Zhang, P., Wu, J., Wang, W., Wang, D., Wang, M., Liu, J., Lin, X., Zhang, J. X., Wang, Z., Yu, Y., &Zhang, Y. F. (2024). Neural substrates for regulating self-grooming behavior in rodents. Journal of Zhejiang University. Science. B, 1-16. Advance online publication. https://doi.org/10.1631/jzus.B2300562 5. Additional detail on animal numbers: please add the number of pregnant dams used, how many of the behavioral animals were from how many different litters, specify that the electrophysiology experiments selected only one animal from each litter (from what I read in the prior review response), and when reporting the number of neurons recorded from each group, break that down by animal. Also, pg. 20 says 12 control rats were used for the electrophysiology but doesn't specify the number of VPA rats, and then pg. 38 says "most VPA-treated animals (n = 10) and some control animals (n = 5) used for behavioral confirmation of the VPA model were also recorded under anesthetized conditions..." If only 5 of the control animals used for behavior were also used for electrophysiology, but 12 control rats were used for electrophysiology, does that mean there is a mix of animals that did and did not undergo behavioral testing that then went on to undergo electrophysiology? R.

We have modified this section and clarified the confusion. The answer to the question is yes, not all the control animals used for behavioral assessments underwent electrophysiological recordings. We have now included this clarification in the main text and the number of neurons for each group of animals (control, VPA, and PV+Cree-Casp) and the number of pregnant rats used for this work.

6. Electrophysiology results details: perhaps this is only because I'm not an electrophysiology expert, but I found these results, particularly for figures 6 and 7, very dense with methodological detail that made it hard to pull out the functional meaning. I would consider moving some of the information about the analyses used to the methods, which might make it easier to find in the results what is functionally being represented by the various measures reported (e.g. what the silhouettes, clusters, patterns, types signify).

R.

We have moved some of the technical descriptions related to the analytical tools to the methods section. And we have modified figure 7 removing the data from PV+Cre animals.

7. As a prior reviewer pointed out, it's unexpected that the counts for FSIs aren't reduced in the VPA group (Fig. 9). I think this should be mentioned in the discussion in the new caveat section where the authors discuss the idea of reduction in expression of PV vs number of PV+ neurons.

R.

We agree with the reviewer, in fact, our interpretation of the data was changed after this comment from the first round of revisions and after performing the experiments with PV+Cre animals in response to those comments. We are now including the following arguments in the discussion section: "However, previous studies have reported that PV+ neuron hypoactivity may be related to a decrease in PV protein expression and maybe related with ASD (Filice 2016; Donato et al., 2013, Contractor et al, 2021). In our data, the decreased levels in PV+ (Fig. 3) were not accompanied by a decrease in the prevalence of neurons with sharp spike waves shapes, typically associated to FSI PV+ interneurons (Fig. 9), opening the possibility that our data could be better explained by a decreased in the levels of PV protein, and not an absolute decrease in the number pf PV+ neurons. This possibility was explored by experimentally depleting PV+ neurons in a transgenic rat model (Fig. 9). This manipulation induced decrease in both, the levels of PV+ neurons and the prevalence of neurons expressing sharp spike waves. This data suggests that in the VPA model an imbalance in the basal ganglia interneuron functionality is related to atypical somatosensory processing" 8. I don't think the authors address Fig. 10 in the discussion. I think it would be worth discussing that the putative FSIs don't correlate with these behaviors but that the MSNs do and contextualizing that in the literature.

R.

The reviewer raised an important concern, and we agree, the idea was not appropriately discussed. We are now including the following paragraph in the discussion: "... our data suggest that the interneuron imbalance would impact on sensory-evoked network activity in the dorsolateral striatum. Since striatal interneurons wont project outside the striatum, the behavioral outcome associated to these effects must implicate the main striatal neurons, MSNs, and their canonical connectivity within the BG (i.e. direct and indirect pathways). Here, our correlation analysis between the proportion of subpopulation of striatal neurons and behavioral parameters (Fig. 10) suggests a relationship between the number of putative MSNs (Type 1) and grooming behavior. However, while in our dataset we did not evaluate the levels of MSNs, diminishing the scope of our interpretations, these data confirm that linking neural dynamics and behavioral outcomes associated to the VPA model would require a wider approach where all the elements of striatal microcircuit are taken into consideration." Minor:

1. In the methods, explicitly state the rationale for exclusive use of male animals R.

Because autism occurs with a ratio of 3:1 higher incidence in males than females (Loomes et al, 2017), the present study was conducted only in male rats. This justification is now included in the methods section.

2. Literature on VPA models &glia in striatum: there is a very recently published paper, see Saavedra-Bonilla et al., Neurochem Res, 2024.

R.

We included the suggested reference.

3. Statement at the end of behavior section on statistics is repeated in the statistics section. I would just keep it in the statistics section and delete from the behavior section.

R.

Corrected as suggested.

4. The authors oscillate between including and not including the "+" for "PV" (vs "PV+") and ChAT (vs "ChAT") inconsistently. The "+" should be there for when referring to it as a marker, such as a "PV+ cell", whereas when referring to the protein itself, the + should not be included.

R.

Corrected 5. In several places in text and figures the authors write "AVP" instead of "VPA" R.

Corrected 6. I think Fig 6G is meant to have an asterisk to indicate significance R.

Corrected 7. On page 33 line 681 I think it is a typo when the authors say "...contrary to S1 animals, control animals..." R.

Corrected 8. Line 770: typo "bee" for "be" R.

Corrected Reviewer 2.

This manuscript investigates striatal interneurons in a rat model of ASD induced by prenatal exposure to valproic acid (VPA). The authors validate the animal model through relevant behavioral assays, quantify interneuron numbers using immunohistochemistry, and conduct electrophysiological recordings in both striatal and cortical regions. The paper has undergone extensive revision, with the authors incorporating a new set of experiments in which they selectively ablated PV neurons using a Cre/Caspase-based strategy. Additionally, the authors have refined some of their claims to better align with the data presented. I have only a few minor comments:

Figure 7L: The population dynamics in the control group appear to have lost the organized rotational trajectories that followed the same path initiated by each stimulus in the train, as previously observed in Figure 6L. The authors could address this discrepancy in their discussion.

R.

Reviewer is correct, we reported this in the results section, but now we are highlighting these concepts in the discussion as follows: "In the control animals, S1 cortical population dynamics presented clear rotational trajectories (Fig. 6L-O), while striatal dynamics presented smaller amplitudes in the rotational component (Fig. 7L-O). Being a primary somatosensory region, the high amplitude in the rotational trajectories observed in S1 is explained by the robustness in representing each stimulus of the train, with virtually all neurons expressing one of the four oscillatory response patterns depicted in Fig. 6J-K. On the other hand, the lower amplitude in DLS is explained by the fact that in this region an important percentage on neurons (about 30 %) did not express a clear response to the somatosensory stimulation (Pattern type 6, Fig. 7J, K)" Lines 652, 654, 658: The term "AVP group" should be corrected to "VPA group." R.

Corrected
